# Establishment and Comprehensive Analysis of Underlying microRNA-mRNA Interactive Networks in Ovarian Cancer

**DOI:** 10.1155/2022/5120342

**Published:** 2022-03-10

**Authors:** Gengchen Ye, Shuyue Feng, Yufei Yang, Zhengzhi Cao, Beilei Zhang, Fu Wang

**Affiliations:** ^1^School of Basic Medical Sciences, Xi'an Jiaotong University, Xi'an 710061, China; ^2^Department of Obstetrics and Gynecology, Tangdu Hospital, Air Force Medical University, Xi'an, China; ^3^School of Pharmacy, Shaanxi Institute of International Trade & Commerce, Xi'an 712046, China

## Abstract

**Background:**

The rate of ovarian cancer (OC) is one of the highest in women's reproductive systems. An improperly expressed microRNA (miRNA) has been discovered to have a vital role in the pathophysiology of OC. However, more research into OC's miRNA-message RNA (mRNA) gene interaction network is required.

**Methods:**

Firstly, the microarray data sets GSE25405 and GSE119055 from the GEO (Gene Expression Omnibus) database were downloaded and then analyzed with the GEO2R tool aiming at identifying DEMs (differential expressed miRNAs) between ovarian malignant tissue and ovarian normal tissue. The whole consistently changed miRNAs were then screened out to be candidate DEMs. For estimating underlying upstream transcription factors, FunRich was employed. miRNet was utilized to determine putative DEMs' downstream target genes. The R program was then used to do the GO annotation as well as the analysis of KEGG pathway enrichment for target genes. The PPI (protein-protein interaction), as well as the DEM-hub gene networks, were created by the Cytoscape software and STRING database. Finally, we chose the GSE74448 dataset to test the precision of hub gene expressions.

**Results:**

We have screened out six (five upregulated and one downregulated) DEMs. The majority of upregulated and downregulated DEMs are likely regulated by SP1 (specificity protein 1). SP4 (s protein 4), POU2F1 (POU class 2 homeobox 1), MEF2A (myocyte-specific enhancer factor 2A), ARID3A (AT-rich interaction domain 3A), and EGR1 (early growth response 1) can regulate upregulated and downregulated DEMs. We have found 807 target genes (656 upregulated and 151 downregulated DEM), being generally enriched in focal adhesion and proteoglycans in cancer, gastric cancer, hepatocellular carcinoma, as well as breast cancer. The majority of hub genes are projected to be controlled by hsa-miR-429, hsa-miR-140-5p, hsa-miR-199a-5p, and hsa-miR-199a-3p after the DEM-hub gene network was built. VEGFA (vascular endothelial growth factor A), EZH2 (enhancer of zeste 2 polycomb repressive complex 2 subunit), and HIF1A (hypoxia inducible factor 1 subunit alpha) expressions are consistent with the GSE74448 dataset in the first 18 hub genes.

**Conclusion:**

We have built an underlying miRNA-mRNA interacting network in OC, giving us unparalleled insight into the disease's diagnosis and treatment.

## 1. Introduction

OC is a malignancy in the female reproductive system, which is prevalent in patients over the age of 50. OC incidence has grown dramatically in Eastern Europe/Southern Europe and Asia in recent years [1]. Patients often show nonspecific pelvic or abdominal symptoms. Nowadays, carbohydrate antigen 125 (CA125) is the most widely utilized and concerning biomarker in OC. Nevertheless, CA125 is less reliable in the diagnosis and not capable of screening early ovarian cancer. In 2009, a randomized clinical trial in China showed that the routine surveillance of OC using the CA125 assay will increase the usage of chemotherapy, reduce the living quality of patients, and can't improve the survival rate of patients [2]. The diagnosis of OC is so difficult as symptoms, medical history, and tests are usually not specific for OC. Because of its asymptomatic development, the disease is often diagnosed as advanced with poor treatment effects, and most patients will develop relapse, acquired drug resistance, and chemotherapy resistance. New early diagnosis and treatment methods are in urgent need.

miRNA, whose size is approximately 22 nucleotides, belongs to the group of small ncRNAs (noncoding RNAs). It regulates one-third of the genomes and downregulates the expression level of its target gene at the posttranscriptional stage as an antisense RNA. miRNA has been linked to a variety of human disorders, and it is thought to be the underlying target for cancer and other diseases detection and treatment [3]. Serum miRNA profiles are a potential and highly reliable biomarker for early detection and diagnosis of OC which has been proven in recent research [4]. miRNA might also operate as a wide regulator of gene expression, influencing particular target mRNA while also modulating the expression of several other genes. In tumor tissues, miRNA can be upregulated or downregulated, while a large proportion of miRNA seems to be upregulated in cancer [5]; for instance, researchers have demonstrated that members of the miR-200 family play an important role in the stress response of the human body and ovarian cancer formation [6]. Exosomal has-miR-21 inhibits ovarian cancer cell apoptosis in ovarian cancer cells while also conferring chemoresistance to cells by binding to the novel target APAF1 (apoptotic peptidase activating factor 1) [7]. Thus, miRNA may be useful as a noninvasive biomarker to improve the accuracy of ovarian cancer screening.

Although the miRNA-mRNA network has been explored in chronic obstructive pulmonary disease (COPD), there has not been an extensively elucidated miRNA-mRNA gene regulatory network in OC[8]. In our study, we analyzed 47 OC samples and 10 normal samples from two datasets (GSE25405 and GSE119055) from GEO in order to find DEMs in OC tissue with normal tissue, and 6 invariably changing miRNAs were filtered out. miRNet has successfully predicted the downstream target genes. The DEM target genes, DEM transcription factors, the DEM-hub gene network, as well as target gene function analysis have all been identified. Furthermore, in the GSE74448 dataset, the tissue level of the hub genes was evaluated and verified. In summary, we set out to discover particular targets for the diagnosis and treatment of OC by establishing an underlying miRNA-mRNA regulation network in the disease.

## 2. Methods

### 2.1. miRNA Microarray

**Figure 1:**
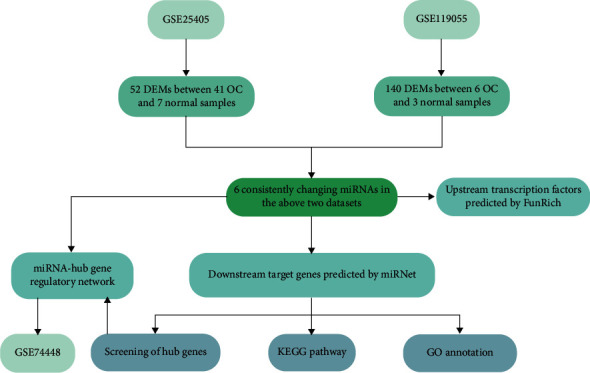
Flow chart of the construction of the miRNA-mRNA regulatory network in OC tissue.

To screen out appropriate miRNA datasets from the GEO (https://www.ncbi.nlm.nih.gov/geo/), our team has set four keywords: “Home sapiens” (organism), “noncoding RNA profiles by array” (study type), “OC” (study keywords), and “tissue” (attribute name). Eventually, two miRNA datasets (GSE25405 and GSE119055) were chosen for subsequent analysis, respectively, on the strength of the GPL7731 platform and the GPL21572 platform. The study design is depicted in Figure 1, and additional details on all the datasets are listed in Table 1.

### 2.2. The Screening of DEMs

GEO2R (http://www.ncbi.nlm.nih.gov/geo/geo2r/), an interactive web program for comparing at least two sets of samples in a GEO series in order to find DEMs in the same experimental conditions, was our choice. To detect DEMs in our investigation, we used log fold change (FC) <−2 or log fold change (FC) > 1.5 as well as a *P*value <0.05 as criteria. The overlap of DEMs in the two datasets (GSE25405 and GSE119055) was analyzed using a Venn diagram, and these invariably changing miRNAs were identified as DEM candidates. The heatmaps of DEM candidates, as well as DEMs, are all created by the R program [9–18].

### 2.3. The Prediction of Transcription Factors Upstream of DEMs

A stand-alone software application FunRich (http://www.funrich.org/) was employed for forecasting underlying upstream transcription factors for potential DEMs. We chose it to study functional enrichment and protein-gene interaction networks. Statistical significance is determined by a *P*value below 0.05.

### 2.4. The Prediction of Downstream Target Genes for DEMs

miRNet (https://www.mirnet.ca/) is a sophisticated toolkit that offers details on miRNA-target interactions as well as the ability to display the interactions in graphical networks. It is frequently used to anticipate downstream target genes of potential DEMs.

### 2.5. The GO and KEGG Analysis of Target Genes

For determining the biological function of DEM target genes, we employed *R* software to perform GO and KEGG pathway analysis. Statistical significance is determined by an adjusted *P*value below 0.05.

### 2.6. The Construction of PPI Network and Screening of Hub Genes

We create a PPI (protein-protein interaction) network of DEM target genes employing STRING (http://stringdb.org/), then visualize it by using the Cytoscape software (version 3.9.0) and Cytoscape plug-in components, and sort nodes per the characteristics of the preloaded PPI network utilizing various topology algorithms. The hub gene (the top 10 nodes in the PPI network) is then screened out by using MCC (maximal clique centrality), which is substantially better than other methods for estimating the precision of essential proteins. Where *S* (v) represents the set of the biggest group including *v* and the product of all positive integers is smaller, the proportion of MCC on node *v* is defined as.

### 2.7. Expression Analysis of Hub Gene by GSE74448 Data Set

GSE74448, based on the platform of GPL18348 from the GEO database, was chosen to perform the analysis of the expressions of hub genes. In this dataset, we have picked up 29 OC and 11 normal tissues. Then, we performed the Student's *t*-test to screen out DEGs between OC and normal tissue. We have set to criteria, which significant hub genes must meet: one is that the upregulated DEM target genes are downregulated, or the downregulated DEM target genes are upregulated; the other is that the *P*value must be below 0.05.

## 3. Results

### 3.1. Identification of DEMs in OC Tissue

**Figure 2:**
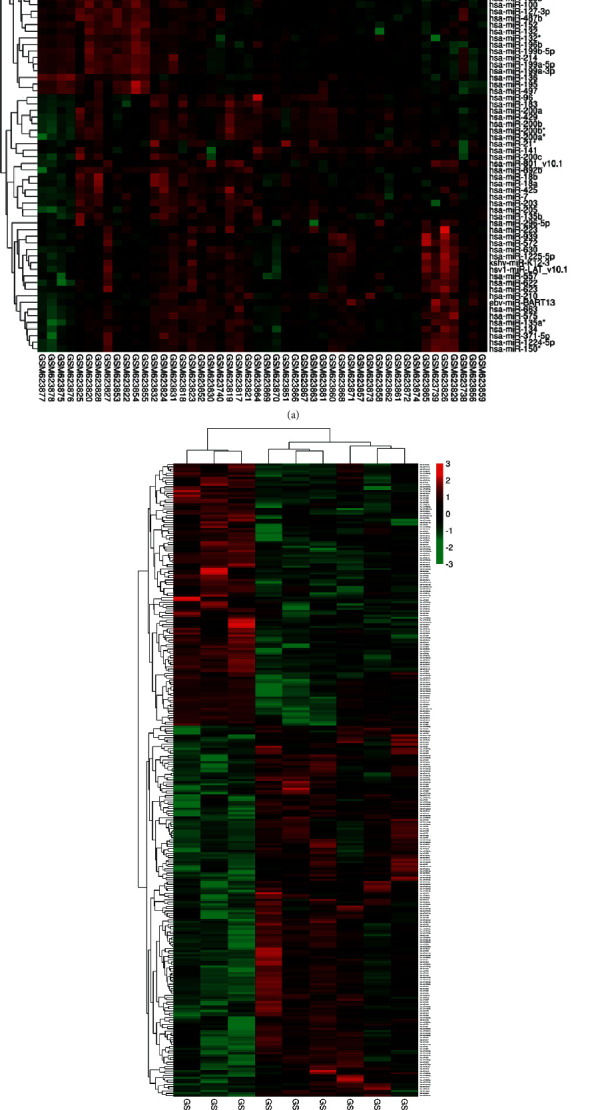
The result of identifying candidate DEMs. (a) GSE25405 dataset; (b) GSE119055 dataset.

**Figure 3:**
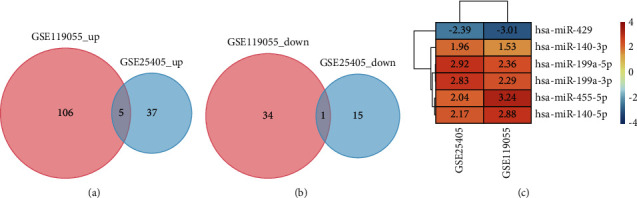
The result of identifying candidate DEMs. (a, b) The overlap of DEMs in the two datasets (GSE25405 and GSE119055) was represented using a Venn diagram. (c) The candidate DEMs' log fold change (FC) heatmap. The screening criteria for DEMs are that a *P* value is less than 0.05 and a log fold change (FC) is more than 1.5 or less than -2. Upregulated DEMs are shown in red, whereas downregulated DEMs are represented in blue. The numbers in the box are the log fold change (FC) values.

In the GSE25405 dataset, 52 DEMs, including 37 upregulated and 15 downregulated, were identified after screening out under the standard of a *P*value 0.05 and log fold change (FC) > 1.5 or log fold change (FC) -2. In the GSE119055 dataset, 140 DEMs, including 106 upregulated and 34 downregulated, were identified after screening out under the standard of a *P*value 0.05 and log fold change (FC) > 1.5 or log fold change (FC) −2. Figures 2(a) and 2(b) show the heatmaps of DEMs, and Supplementary Tables S1 and S2 show the specific information about DEMs. Among these DEMs, five upregulated (has-miR-199a-3p, has-miR-140-3p, has-miR-199a-5p, has-miR-140-5p, and has-miR-455-5p) and one downregulated (has-miR-429) were invariably changed in both two datasets (Figures 3(a)–3(c)).

### 3.2. Prediction of Upstream Transcription Factors of DEMs

**Figure 4:**
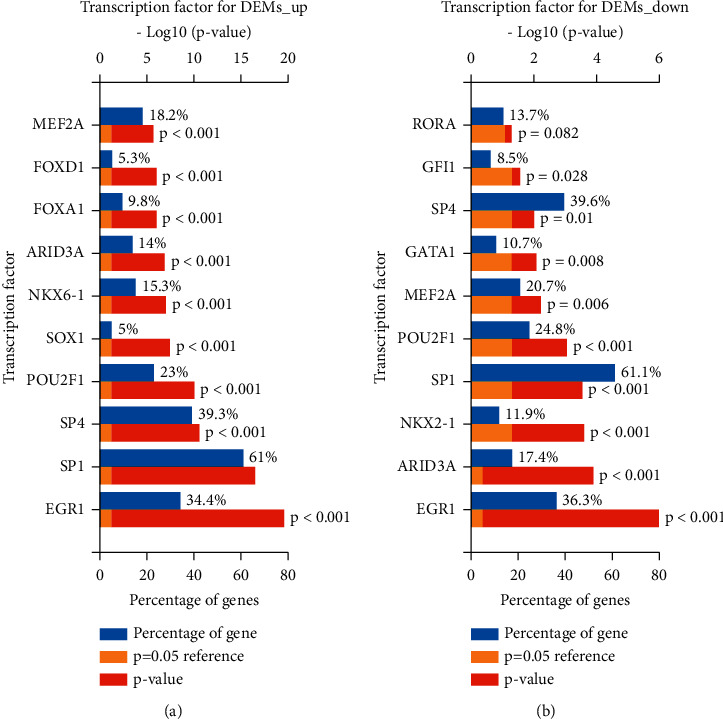
miRNet predicts underlying transcription factors of DEMs. (a) Transcription factors for DEMs that are upregulated; (b) transcription factors for DEMs that are downregulated.

We employed a software named FunRich to forecast upstream transcription factors among candidate DEMs. Figures 4(a) and 4(b) are transcription factors that upregulate and downregulate DEM. The transcription factors were SP1, RORA (RAR related orphan receptor A), SP4, GATA1 (GATA binding protein 1), GFI1 (growth factor independent 1 transcriptional repressor), NKX2-1(NK2 homeobox 1), MEF2A, POU2F1, ARID3A, and EGR1 for downregulated DEMs. Concerning upregulated DEMs, transcription factors were NKX6-1 (NK6 homeobox 1), SP1 (specificity protein 1), SP4, EGR1, SOX1 (SRY-Box transcription factor 1), ARID3A, FOXA1 (forkhead box A1), POU2F1, FOXD1 (forkhead box D1), and MEF2A.

### 3.3. Prediction of Downstream Target Genes of DEMs

**Figure 5:**
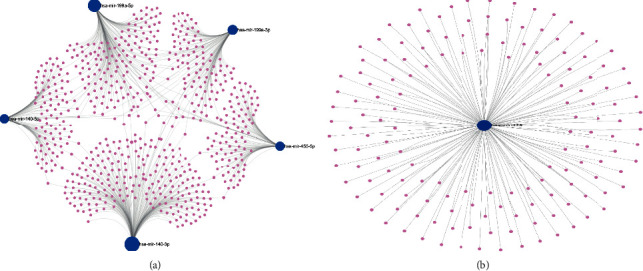
miRNet predicts the underlying transcription factors of DEMs. (a) The miRNA-target gene network for DEMs that have been upregulated. (b) The miRNA-target gene network for DEMs that have been downregulated.

In all 865 candidate DEM target genes were found by the miRNet database (704 upregulated DEM target genes as well as 151 downregulated DEM target genes).  Figure 5(a) and 5(b), respectively, show the upregulated, as well as downregulated DEMs, and the target genes of them, are represented in the DEM target gene network. Moreover, Table 2 shows the amount of target genes per DEM. Supplementary Table S3 shows the predicted target genes.

### 3.4. GO (Gene Ontology) and KEGG Analysis of Target Genes

**Figure 6:**
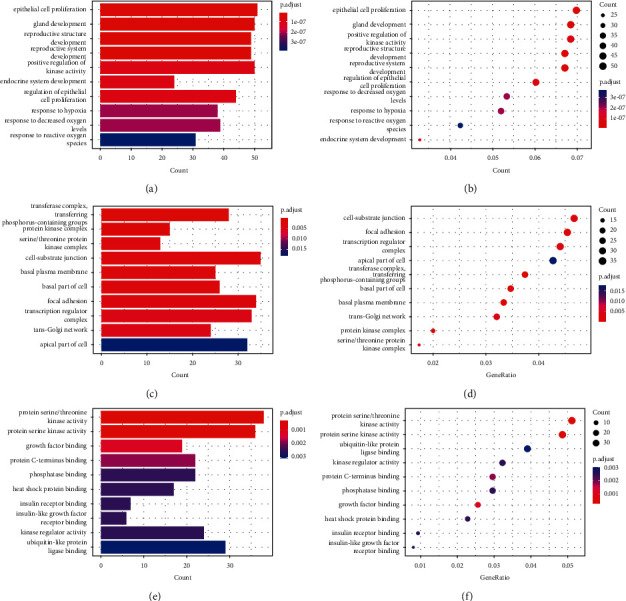
(a–f) GO annotation study for DEM target genes in the biological process, cellular component, and molecular function. (a,c, and e) Barplot; (b, d, and f) dotplot. Significantly altered GOs were determined by the adjusted *P* value below 0.05. The adjusted *P* value is shown on the *x*-axis. The *y*-axis displays the GO annotation terms (a, c, and e), as well as the gene ratios (b, d, and f) of each term.

On 865 DEM target genes, we have carried out GO and KEGG analysis. DEM target genes are abundant in epithelial cell proliferation, gland development, and positive regulation of kinase activity, according to biological process analysis (Figures 6(a)–6(f)), particularly; DEM target genes are mostly found at the cell-substrate junction and focal adhesion, according to cellular component analysis, and transcription regulator complex; DEM target genes are considerably enriched in protein serine/threonine kinase activity, protein serine kinase activity, and ubiquitin-like protein ligase binding, according to molecular function study.

**Figure 7:**
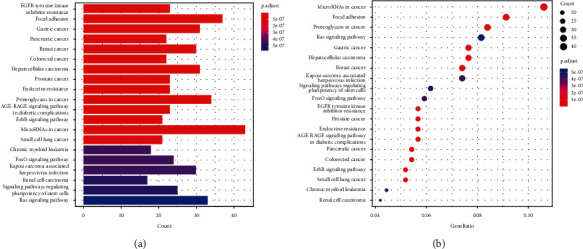
(a, b) The target genes of DEMs were analyzed using the KEGG pathway database. Significantly altered routes were determined by the adjusted *P* value below 0.05. The corrected *P* value (a) and gene ratio (b) of each phrase are displayed on the *x*-axis, while the KEGG pathway terms are displayed on the *y*-axis.

KEGG pathway analysis shows that DEM target genes are significantly enriched in focal adhesion, proteoglycans in cancer, gastric cancer, hepatocellular carcinoma, and breast cancer (Figures 7(s) and 7(b)).

### 3.5. The Construction of PPI and DEM-Hub Gene Network

**Figure 8:**
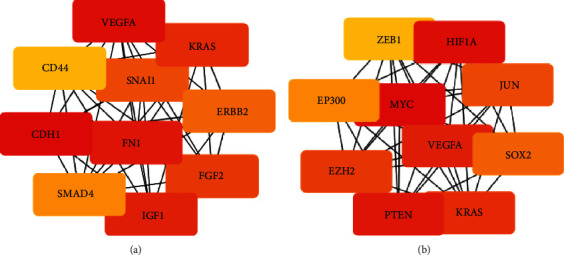
The results of distinguishing the hub genes of DEMs by PPI network. (a) Upregulated PPI network in DEMs for the first 10 hub genes. (b) Downregulated PPI network in DEMs for the first 10 hub genes.

We used the STRING database to create a PPI (protein-protein interaction) network comprising DEM-targeted genes, which are upregulated and downregulated. Then, to screen out Hub genes, we used the Cytoscape and cytoHubba plugins. The first 10 hub genes, which are upregulated and downregulated in DEMs, are all shown in Figures 8(a) and 8(b). Concerning upregulated DEMs, the top 10 hub genes are MET (MET proto-oncogene and receptor tyrosine kinase), VEGFA, HIF1A, MTOR (mammalian target of rapamycin), GSK3B (glycogen synthase kinase 3 beta), SOX2 (SRY-box transcription factor 2), CDH2 (cadherin 2), IGF1R (insulin-like growth factor 1 receptor), FGF2 (fibroblast growth factor 2) and KDR (kinase insert domain receptor); and the top 10 hub genes for downregulated DEMs were MYC (MYC proto-oncogene and BHLH transcription factor), HIF1A, PTEN (MMAC1, mutated in multiple advanced cancers 1), VEGFA, KRAS (V-Ki-ras2 Kirsten rat sarcoma viral oncogene homolog), EZH2, JUN (Jun proto-oncogene and AP-1 transcription factor subunit), SOX2, EP300 (E1A binding protein P300) and ZEB1 (zinc finger E-box binding homeobox 1) (Table 3).

**Figure 9:**
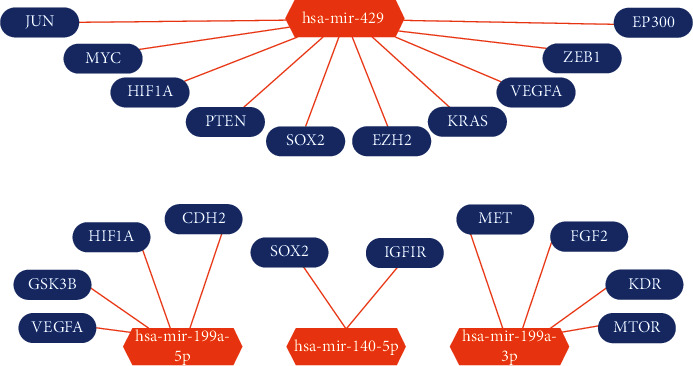
The regulatory network of identified miRNA-hub genes. (a) The PPI network of the first 10 central genes was upregulated in DEMs. (b) The PPI network of the first 10 central genes was downregulated in DEMs.

We used Cytoscape software to create a DEM-hub gene network to comprehend the molecular mechanism of the DEMs in OC tissue. The following are the relations between upregulated DEMs and hub genes: VEGFA, GSK3B, HIF1A, and CDH2 were associated with hsa-miR-199a-5p; hsa-miR-140-5p interacted with two hub genes, SOX2 and IGF1R; and hsa-miR-199a-3p interacted with MET, FGF2, KDR, and MTOR. Downregulated DEMs made interactions with 10 hub genes, including JUN, MYC, HIF1A, PTEN, SOX2, EZH2, KRAS, VEGFA, ZEB1, and EP300 (Figure 9).

### 3.6. Hub Genes Expression Validation

**Figure 10:**
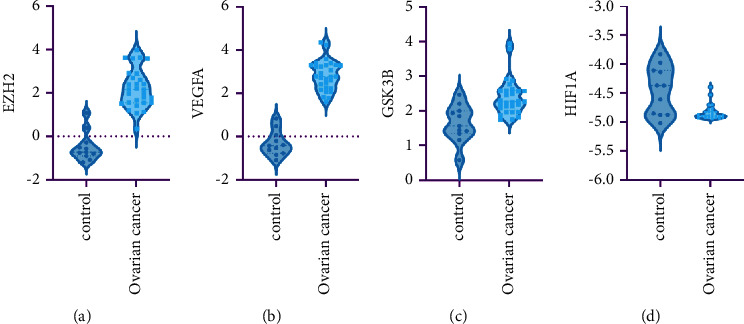
The top 4 hub genes' mRNA expression levels based on the dataset GSE74448. (a) EZH2; (b) VEGFA; (c) GSK3B; and (d) HIF1A.

The GSE74448 dataset was employed for verifying tissue levels of the top four hub genes (EZH2, VEGFA, GSK3B, and HIF1A) based on the DEM-hub gene network. For downregulated DEMs, only the HIF1A expression surged in OC tissue (Figure 10(d)). Compared with normal tissue, the expression of upregulated DEMs (VEGFA and EZH2) was increased in OC tissue (Figures 10(a) and 10(b)). Therefore, hsa-miR-429-VEGFA, hsa-miR-429-EZH2, and hsa-miR-199a-5p-HIF1A were verified as three underlying regulatory pathways in OC tissues.

## 4. Discussion

In recent years, many studies on the diagnosis and treatment of OC have been carried out. However, the understanding of the underlying molecular biology pathogenesis of OC is still unclear, so the treatment effect of ovarian cancer patients is still poor. Through microarray technology, thousands of gene changes in the process of occurrence and development of various diseases can be revealed more easily, so as to construct an effective miRNA-mRNA gene regulatory network. In our study, we extracted data from two data sets, namely, GSE25405 and GSE119055, to distinguish DEMs between OC tissue and normal tissue.

Five upregulated DEMs (hsa-miR-455-5p, hsa-miR-199a-5p, hsa-miR-140-5p, hsa-miR-199a-3p, and hsa-miR-140-3p) and one downregulated DEM (hsa-miR-429) are constantly changing in the two data sets and have been identified as the candidate DEMs for further analysis. Both strands (-5p and -3p) of has-miR-455 target the same genes to regulate articular cartilage homeostasis, with underlying treatment value for osteoarthritis (OA), are upregulated by Sox9 (SRY-box transcription factor 9), a crucial transcription factor of cartilage differentiation and function [19]. has-miR-199a-5p is the key regulator of the abnormal *α*1-antitrypsin (AAT)-deficient monocyte unfolded protein response, which can be regulated by epigenetic silencing in chronic obstructive pulmonary disease [20]. has-miR-140-5p is an underlying prognostic factor for gastric cancer patients, being capable of mediating YES1 (YES proto-oncogene 1 and Src family tyrosine kinase) suppression [21]. It has appeared that the expression level of has-miR-199a-3p is concerned with acute heart failure (AHF) [22]. has-miR-140-3p downregulates the oncogene BRD9 (bromodomain containing 9) expression in squamous cell lung cancer (SqCLC) and inhibits SqCLC tumorigenesis, thus may become a new inhibitor of SqCLC [23]. As a member of the has-miR-200 family (composed of 5 miRNAs: has-miR-141, has-miR-200a, has-miR-200b, has-miR-200c, and has-miR-429), the expression of has-mir-429 is in connection with the survival rate of liver cancer [24]. It is worth noting that many of these miRNAs have been reported as underlying treatment targets for many human diseases, and their mechanism of action in OC requires further research to clarify more clearly.

In recent years, more and more research has revealed the fact that transcription factors are capable of regulating the expression of miRNA [25]. Therefore, we forecast that this process could potentially take place. SP1 (specific protein 1), a promoter-specific binding factor, has been proven to be overexpressed in a variety of cancers, and in connection with poor prognosis, downregulation of SP1 regulatory genes has also been shown to be drug-dependent. It is expected to account for the highest proportion of upregulated as well as downregulated DEM and has been widely studied in OC. Studies have shown that the new signal axis of has-miR-141/KLF12 (Kruppel-like factor 12)/SP1/survivin can enhance anoikis drug resistance, which is promising as a feasible therapeutic target for transmutative OC [26]. EGR1 is expected to regulate the upregulation and downregulation of DEM expression. Studies related to clinical cases have indicated that the expression levels of EGR1, as well as has-miR-152 in OC tissues, are significantly reduced, while EGR1-has-miR-152 downregulation of ATG14 (autophagy related 14) can make OC cells obtain cisplatin-induced apoptosis sensitivity by restraining cytoprotective autophagy [27].

The conclusion that DEMs are substantially enriched in focal adhesion, proteoglycans in cancer, and endocrine resistance is mainly indicated by the structure analyzed by KEGG. During cell migration, the dynamic regulation of focal adhesion formation and disassembly plays a crucial part in the metastasis of vast cancers including OC. SOCE (store-operated Ca entry) inhibitors significantly weaken the assembly and disassembly of focal adhesions. Therefore, SOCE may be a feasible medicinal target for OC [28]. The syndecans are a protein family formed by the joint combination of four single transmembrane domain proteins. Members can interact with plenty of ligands, including fibroblast growth factors, TGF-*β* (transforming growth factor-*β*), VEGF (vascular endothelial growth factor), and other molecules, and usually carry 3 to 5 heparan sulfate and chondroitin sulfate chains. The CD44 (CD44 molecule (Indian blood group)) receptor, a characteristic biomarker of ovarian cancer stem cells, is a group of intact membrane proteins with high sugar content, wide distribution, and a molecular weight of (85–160) × 10kD [29]. Endocrine therapy has been recognized as an effective way to treat chemically resistant ovarian cancer. Studies have shown that targeting the autocrine IL-6 (interleukin-6)-SPINK1 (serine peptidase inhibitor Kazal type) signal axis can inhibit the metastasis and spread of ovarian clear cell carcinoma, which provides a possibility while replicating the therapy. Therefore, inhibition of SOCE or syndecan-1, promoting CD44 receptor expression, and induction of the targeting autocrine IL-6-SPINK1 signal axis may represent a new treatment strategy for OC.

GO analysis results also have important reference values. In terms of molecular function (MF), ubiquitin-like protein ligase binding kinase regulatory activity, protein C-terminal binding, phosphatase binding, and growth factor binding have been shown to exhibit a significant correlation with OC. BRCA1 (breast cancer gene 1) is a nuclear phospholipid, which inhibits breast cancer and ovarian cancer tumors. It is usually combined with BARD1 (BRCA1 associated RING domain 1) to form an annular isoprenoid, acting as a ubiquitin (Ub) ligase. Its activity is greatly increased, and it contains a special domain that is interrupted by mutations that are susceptible to cancer [30]. CTBP2 (C-terminal binding protein 2) was found to be increased in epithelial ovarian carcinoma (EOC). Many research studies have demonstrated that CTBP2 and CTBP1 (C-terminal binding protein 1) have an extremely important role in suppressing the expression and activity of DR4/5 (death-receptors 4/5), and may maintain the possibility of CTBP as a high-grade serous ovarian cancer (HGSOC) treatment strategy [31]. In the pathological angiogenesis of female ovarian cancer, the expression level of vascular endothelial cadherin (VEC) and the expression of claudin-5 and vascular endothelial-protein tyrosine phosphatase are lowered in parallel [32]. TGF-*β* signaling is a considerable component in gene regulation in cancer. The TGF-*β* signal tends to increase in the late tumor malignant stage. Studies have shown that the expression of nc886 (a 101-nucleotide (nt)-long ncRNA) is induced by TGF-*β*, then nc886 is combined with dicer (an RNase III endonuclease) to inhibit miRNA maturation [33].

In terms of cellular components (CC), transcriptional regulatory complexes have been elucidated to play an essential part in the incidence and progression of OC. The overexpression of the oncogene MYBL2 (also known as B-Myb, MYB roto-oncogene like 2) has been confirmed to be associated with an increased rate of cell proliferation and can also become a valuable biomarker for poor cancer prognosis. However, the interaction of B-Myb with the MuvB (multivulval B, also known as LINC (LIN complex)) core complex in proliferating cells culminates in the formation of the product MMB (Myb-MuvB) complex, a process that is also critical for the transcription of genes required for mitosis promotion. Decreased or even loss of DREAM (drosophila, RB, E2F, and Myb) target gene repression in breast and ovarian cancer has been likewise demonstrated to be associated with overexpression of B-Myb. Based on this mechanism, targeting B-Myb to restore the body's control of the cell cycle may serve as a more effective and novel approach to treating not only ovarian cancer but other cancers as well [34].

In the biological process (BP), the positive regulation of kinase activity and hypoxia in the incidence and progression of OC has also been clarified to play a very essential role. To begin with, GSK-3 (Glycogen synthase kinase 3) can participate in the proliferation of female ovarian cancer cells by accelerating the expression level of cyclin D1, which is expected to be an explorable and therapeutic direction for OC [35]. The oxygen dependence of HIF1A protein works in the degradation and regulation of the activity of the transcription factor HIF (hypoxia-inducible factor), which is an important regulatory process in the hypoxia response. Past studies have demonstrated that the gene expression characteristics of hypoxia response are firm predictors of the clinical outcome of ovarian cancer and breast cancer and have an extremely important relationship with the significantly poorer prognosis of ovarian cancer and breast cancer [36].

By constructing the DEM-hub gene network, most of the hub genes may be targeted by hsa-miR-429, hsa-miR-199a-3p, hsa-miR-199a-5p, and hsa-miR-140-5p. In the first 18 central genes, the expression of only three genes (EZH2, VEGFA, and HIF1A) is consistent with the expression in the GSE74448 dataset, which may be due to the fact that they come from different sample sources. One of the epigenetic modifiers, EZH2 has been studied to promote ovarian cancer chemoresistance and recurrence [37]. VEGFA is a widely recognized treatment target of OC [38]. What we can know from the research is that has-miR-6086 can directly or indirectly downregulate the OC2 (onecut2)/VEGFA/EGFL6 (EGF-like domain multiple 6) axes to inhibit the angiogenesis network in OC, and may become a new research hotspot as an underlying target for tumor therapy [39]. HIF1A is a transcription factor that responds to hypoxia. By studying the transcription of inducible HIF1A-AS2 (hypoxia inducible factor 1 subunit alpha antisense RNA 2), there is hope for a new method for ovarian cancer treatment that combines effective anticancer therapy with OC standard-of-care chemotherapy [40].

Although we have uncovered an underlying miRNA-mRNA regulatory network in OC tissue, there were still some limitations in the current study. First, we concentrated on miRNAs and mRNAs only between OC and normal tissues, whereas a few of them may be distinct in different phases of OC. Second, through the prediction of the public database, we found miRNA-mRNA interactions.Further in vivo experiments are required to verify the analysis.

## 5. Conclusion

In conclusion, we have developed three underlying miRNA-mRNA pathways (hsa-miR-429-VEGFA, hsa-miR-429-EZH2, and hsa-miR-199a-5p-HIF1A) in OC tissues as underlying biomarkers for diagnosis and therapy of OC patients based on bioinformatics analysis and the GEO database. Our team truly hopes that all of the discoveries would be useful in further research and in improving the prognosis of patients with OC.

## Figures and Tables

**Table 1:** Details for GEO OC data.

Accession	Platform	Sample	Normal	OC	RNA/microRNA
GSE25405	GPL7731	Tissue	7	41	microRNA
GSE119055	GPL21572	Tissue	3	6	microRNA
GSE74448	GPL18348	Tissue	11	29	RNA

**Table 2:** Underlying target genes of the markedly upregulated as well as downregulated DEMs.

Upregulated DEMs	Number	Downregulated DEMs	Number
hsa-miR-455-5p	99	hsa-miR-429 gene	151
hsa-miR-140-3p	224		
hsa-miR-140-5p	101		
hsa-miR-199a-5p	166		
hsa-miR-199a-3p	114		
Total	704	Total	151

**Table 3:** The top 10 hub genes of the markedly upregulated as well as downregulated DEMs in the PPI network in MCC ranking.

Upregulated DEMs		Downregulated DEMs	
Gene symbol	Score	Gene symbol	Score
MET	9.22337*E* + 13	MYC	4897965
VEGFA	9.22337*E* + 13	HIF1A	4895915
HIF1A	9.22337*E* + 13	PTEN	4895776
MTOR	9.22337*E* + 13	VEGFA	4889019
GSK3B	9.22337*E* + 13	KRAS	4886053
SOX2	9.22337*E* + 13	EZH2	4799694
CDH2	9.22337*E* + 13	JUN	4534688
IGF1R	9.22337*E* + 13	SOX2	4401487
FGF2	9.22337*E* + 13	EP300	4366513
KDR	9.22337*E* + 13	ZEB1	4354588

## Data Availability

The supplementary information files provide the data used to support the findings of this investigation.

## Supplementary Materials

Supplementary Materials
*Table S1.* DEMs between OC and normal tissue from the GSE25405 dataset. *Table S2.* DEMs between OC and normal tissue from the GSE119055 dataset. *Table S3.* Target genes of DEMs predicted by miRNet.
