# Supplementary material for: Establishment and Comprehensive Analysis of Underlying microRNA-mRNA Interactive Networks in Ovarian Cancer
Source: J Oncol. 2022 Mar 10;2022:5120342. doi: 10.1155/2022/5120342 (PMC8930263; doi:10.1155/2022/5120342)
Supplement: Supplementary Materials — Table S1. DEMs between OC and normal tissue from the GSE25405 dataset. Table S2. DEMs between OC and normal tissue from the GSE119055 dataset. Table S3. Target genes of DEMs predicted by miRNet. [file 5120342.f1.zip › 5120342.f1/Supplementary Table S1.pdf]

Table S1 DEMs between OC and normal tissue from GSE25405 dataset.

ID	adj.P.Val	P.Value	t	B	logFC
hsa-miR-99a	2.53E-02	5.86E-03	2.87161	-2.7746	2.34836
hsa-miR-99a	2.75E-02	6.58E-03	2.829139	-2.8793	2.37399
hsa-miR-99a	2.75E-02	6.60E-03	2.827603	-2.8831	2.329534
hsa-miR-99a	2.81E-02	6.78E-03	2.817822	-2.907	2.325603
hsa-miR-99a	2.81E-02	6.80E-03	2.817068	-2.9089	2.336629
hsa-miR-99a	2.83E-02	6.89E-03	2.812128	-2.921	2.334549
hsa-miR-99a	2.83E-02	6.90E-03	2.811553	-2.9224	2.330271
hsa-miR-99a	2.96E-02	7.30E-03	2.790699	-2.9731	2.330066
hsa-miR-99a	5.55E-02	1.67E-02	2.47058	-3.7163	2.209575
hsa-miR-99a	6.20E-02	1.95E-02	2.408225	-3.8529	2.206868
hsa-miR-99a	6.27E-02	1.99E-02	2.40032	-3.87	2.178213
hsa-miR-99a	6.31E-02	2.01E-02	2.397598	-3.8759	2.144291
hsa-miR-99a	6.52E-02	2.09E-02	2.381791	-3.9099	2.123934
hsa-miR-99a	6.53E-02	2.09E-02	2.380631	-3.9124	2.122517
hsa-miR-99a	6.58E-02	2.11E-02	2.376212	-3.9219	2.144406
hsa-miR-99a	7.47E-02	2.53E-02	2.302666	-4.0776	2.089683
hsa-miR-96	1.05E-03	7.65E-05	-4.288605	1.2712	-2.213402
hsa-miR-96	1.05E-03	8.04E-05	-4.273461	1.2233	-2.211239
hsa-miR-96	1.05E-03	8.06E-05	-4.272901	1.2216	-2.227901
hsa-miR-96	1.06E-03	8.23E-05	-4.266797	1.2023	-2.147228
hsa-miR-96	1.08E-03	8.85E-05	-4.244726	1.1327	-2.174318
hsa-miR-96	1.08E-03	9.03E-05	-4.238876	1.1143	-2.202934
hsa-miR-96	1.08E-03	9.07E-05	-4.237492	1.1099	-2.175984
hsa-miR-96	1.13E-03	9.80E-05	-4.213966	1.036	-2.169404
hsa-miR-96	1.13E-03	1.01E-04	-4.206345	1.012	-2.14111
hsa-miR-96	1.13E-03	1.02E-04	-4.201108	0.9956	-2.178772
hsa-miR-96	1.15E-03	1.07E-04	-4.186158	0.9488	-2.153154
hsa-miR-96	1.18E-03	1.13E-04	-4.169669	0.8973	-2.114467
hsa-miR-96	1.36E-03	1.42E-04	-4.102105	0.6871	-2.097294
hsa-miR-96	1.36E-03	1.42E-04	-4.102065	0.687	-2.109547
hsa-miR-96	1.37E-03	1.43E-04	-4.098443	0.6758	-2.109643
hsa-miR-96	1.48E-03	1.59E-04	-4.067311	0.5796	-2.088219
hsa-miR-663	8.35E-04	4.47E-05	-4.448609	1.782	-2.703652
hsa-miR-663	9.60E-04	5.33E-05	-4.396325	1.6142	-2.699316
hsa-miR-663	1.05E-03	7.42E-05	-4.297553	1.2996	-2.640799
hsa-miR-663	1.05E-03	7.48E-05	-4.295122	1.2919	-2.68123
hsa-miR-663	1.05E-03	7.75E-05	-4.284668	1.2588	-2.592667
hsa-miR-663	1.48E-03	1.60E-04	-4.065374	0.5736	-2.439832
hsa-miR-663	1.73E-03	1.92E-04	-4.009379	0.4016	-2.453402
hsa-miR-663	2.95E-03	3.65E-04	-3.808938	-0.2036	-2.548525
hsa-miR-630	5.08E-03	7.34E-04	-3.585104	-0.8587	-2.934175
hsa-miR-630	5.73E-03	8.53E-04	-3.53618	-0.9987	-2.692548

hsa-miR-630	7.49E-03	1.17E-03	-3.430825	-1.2962	-2.972883
hsa-miR-630	7.66E-03	1.21E-03	-3.419872	-1.3269	-2.556843
hsa-miR-622	5.19E-04	1.94E-05	-4.692403	2.5759	-3.29006
hsa-miR-575	6.58E-03	1.00E-03	-3.482381	-1.1513	-2.301284
hsa-miR-497	2.31E-05	2.14E-07	5.954607	6.9025	3.236248
hsa-miR-497	2.31E-05	2.33E-07	5.931803	6.8221	3.29102
hsa-miR-497	2.31E-05	2.41E-07	5.922593	6.7897	3.266449
hsa-miR-497	2.33E-05	2.93E-07	5.86846	6.5991	3.2562
hsa-miR-497	2.33E-05	3.21E-07	5.843498	6.5113	3.27675
hsa-miR-497	2.33E-05	3.29E-07	5.836947	6.4883	3.249822
hsa-miR-497	2.33E-05	3.31E-07	5.835475	6.4831	3.281152
hsa-miR-497	2.82E-05	4.29E-07	5.764236	6.2331	3.27486
hsa-miR-497	8.01E-05	1.45E-06	5.428032	5.0627	3.347563
hsa-miR-497	9.32E-05	1.80E-06	5.36719	4.8528	3.313554
hsa-miR-497	1.21E-04	2.78E-06	5.245488	4.4348	3.470032
hsa-miR-487b	1.45E-02	2.81E-03	3.13452	-2.1018	2.532134
hsa-miR-487b	1.79E-02	3.68E-03	3.039401	-2.35	2.522726
hsa-miR-455-5p	4.00E-03	5.58E-04	3.673563	-0.6026	2.091619
hsa-miR-455-5p	5.47E-03	8.01E-04	3.556385	-0.941	2.140734
hsa-miR-455-5p	7.16E-03	1.11E-03	3.450156	-1.2421	1.954498
hsa-miR-455-5p	8.20E-03	1.31E-03	3.39448	-1.3976	1.952383
hsa-miR-455-5p	1.25E-02	2.27E-03	3.2085	-1.9052	2.075203
hsa-miR-455-3p	2.14E-03	2.45E-04	3.933542	0.1706	2.082989
hsa-miR-455-3p	2.72E-03	3.33E-04	3.838004	-0.1169	2.041117
hsa-miR-455-3p	3.13E-03	4.03E-04	3.777291	-0.2976	2.011701
hsa-miR-455-3p	3.27E-03	4.26E-04	3.759359	-0.3507	2.001349
hsa-miR-455-3p	3.29E-03	4.30E-04	3.756891	-0.358	2.019615
hsa-miR-455-3p	3.34E-03	4.39E-04	3.750503	-0.3769	2.015907
hsa-miR-455-3p	3.59E-03	4.78E-04	3.723023	-0.4578	2.136004
hsa-miR-455-3p	3.88E-03	5.36E-04	3.686753	-0.5641	1.99564
hsa-miR-455-3p	5.56E-03	8.21E-04	3.548509	-0.9635	2.07976
hsa-miR-455-3p	5.97E-03	9.02E-04	3.517869	-1.0508	2.061286
hsa-miR-455-3p	7.00E-03	1.07E-03	3.460098	-1.2141	2.041994
hsa-miR-455-3p	9.51E-03	1.57E-03	3.333853	-1.5651	2.139804
hsa-miR-455-3p	1.74E-02	3.57E-03	3.049778	-2.3231	2.259633
hsa-miR-429	7.24E-05	1.20E-06	-5.480338	5.2436	-2.456537
hsa-miR-429	7.24E-05	1.22E-06	-5.475165	5.2257	-2.439709
hsa-miR-429	7.92E-05	1.37E-06	-5.443599	5.1165	-2.397313
hsa-miR-429	8.01E-05	1.47E-06	-5.422852	5.0448	-2.450823
hsa-miR-429	8.01E-05	1.48E-06	-5.421379	5.0397	-2.418131
hsa-miR-429	8.02E-05	1.52E-06	-5.414952	5.0175	-2.41973
hsa-miR-429	9.68E-05	1.92E-06	-5.349859	4.7931	-2.373828
hsa-miR-429	9.68E-05	1.95E-06	-5.344895	4.776	-2.390403
hsa-miR-429	1.06E-04	2.18E-06	-5.314383	4.6711	-2.346744

hsa-miR-429	1.06E-04	2.22E-06	-5.309179	4.6532	-2.366514
hsa-miR-429	1.07E-04	2.28E-06	-5.301544	4.627	-2.410816
hsa-miR-429	1.14E-04	2.48E-06	-5.277504	4.5445	-2.370102
hsa-miR-429	1.15E-04	2.60E-06	-5.264168	4.4988	-2.363896
hsa-miR-429	1.29E-04	3.07E-06	-5.217773	4.3401	-2.360966
hsa-miR-429	1.42E-04	3.52E-06	-5.179196	4.2084	-2.328723
hsa-miR-429	1.47E-04	3.94E-06	-5.147804	4.1015	-2.34815
hsa-miR-424	4.44E-11	6.28E-14	10.084927	21.3992	4.811093
hsa-miR-424	4.44E-11	7.73E-14	10.025246	21.1996	4.869215
hsa-miR-424	4.44E-11	7.94E-14	10.017648	21.1742	4.819171
hsa-miR-424	4.44E-11	1.11E-13	9.921201	20.8506	4.838647
hsa-miR-424	4.44E-11	1.26E-13	9.885504	20.7305	4.885638
hsa-miR-424	4.44E-11	1.26E-13	9.884573	20.7274	4.821637
hsa-miR-424	4.44E-11	1.28E-13	9.881404	20.7167	4.917572
hsa-miR-424	1.38E-08	4.54E-11	8.246468	15.0671	5.138311
hsa-miR-424	5.41E-08	2.17E-10	7.820638	13.5572	5.205727
hsa-miR-371-5p	3.06E-03	3.87E-04	-3.790497	-0.2585	-2.573689
hsa-miR-34c-5p	6.75E-02	2.20E-02	2.360329	-3.9559	2.289714
hsa-miR-34b*	4.94E-02	1.41E-02	2.539017	-3.5633	1.661409
hsa-miR-34b*	6.27E-02	1.98E-02	2.401906	-3.8666	1.552538
hsa-miR-34b*	6.59E-02	2.12E-02	2.37463	-3.9253	1.573509
hsa-miR-34b*	6.81E-02	2.22E-02	2.356544	-3.9639	1.562182
hsa-miR-34b*	6.94E-02	2.29E-02	2.343903	-3.9908	1.5128
hsa-miR-34b*	7.13E-02	2.38E-02	2.32801	-4.0244	1.568706
hsa-miR-34b*	7.85E-02	2.68E-02	2.278118	-4.1287	1.521983
hsa-miR-34b*	8.39E-02	2.91E-02	2.24251	-4.202	1.519791
hsa-miR-342-3p	3.66E-02	9.32E-03	2.698496	-3.1942	1.526909
hsa-miR-29a	9.33E-03	1.53E-03	3.34276	-1.5406	1.878239
hsa-miR-29a	1.04E-02	1.75E-03	3.295861	-1.669	1.887923
hsa-miR-29a	1.04E-02	1.76E-03	3.295061	-1.6712	1.895419
hsa-miR-29a	1.05E-02	1.80E-03	3.287186	-1.6927	1.880301
hsa-miR-29a	1.19E-02	2.11E-03	3.23257	-1.8405	1.81478
hsa-miR-29a	1.22E-02	2.18E-03	3.222054	-1.8688	1.821982
hsa-miR-29a	1.22E-02	2.19E-03	3.219961	-1.8744	1.831985
hsa-miR-29a	1.82E-02	3.75E-03	3.032058	-2.3689	1.841247
hsa-miR-29a	2.06E-02	4.40E-03	2.97514	-2.5146	1.779915
hsa-miR-29a	2.10E-02	4.51E-03	2.9666	-2.5363	1.809819
hsa-miR-29a	2.20E-02	4.78E-03	2.945331	-2.5901	1.80895
hsa-miR-29a	2.40E-02	5.41E-03	2.900506	-2.7027	1.794263
hsa-miR-29a	2.43E-02	5.58E-03	2.889359	-2.7305	2.121966
hsa-miR-29a	2.75E-02	6.59E-03	2.828204	-2.8816	1.748034
hsa-miR-29a	3.29E-02	8.28E-03	2.743131	-3.0879	1.942102
hsa-miR-29a	4.75E-02	1.33E-02	2.562683	-3.5096	2.149862
hsa-miR-27b	7.16E-04	3.48E-05	4.522549	2.0209	2.518803

hsa-miR-27b	7.28E-04	3.61E-05	4.511516	1.9852	2.575559
hsa-miR-27b	7.95E-04	4.12E-05	4.472372	1.8586	2.440305
hsa-miR-27b	8.13E-04	4.32E-05	4.458823	1.8149	2.464547
hsa-miR-27b	8.67E-04	4.67E-05	4.435375	1.7395	2.460317
hsa-miR-27b	9.54E-04	5.26E-05	4.400446	1.6274	2.483338
hsa-miR-27b	9.63E-04	5.39E-05	4.393074	1.6038	2.515143
hsa-miR-27b	9.76E-04	5.66E-05	4.378302	1.5565	2.46171
hsa-miR-27b	9.85E-04	5.77E-05	4.372865	1.5392	2.518375
hsa-miR-27b	9.85E-04	5.83E-05	4.36962	1.5288	2.487885
hsa-miR-27b	9.85E-04	6.00E-05	4.361173	1.5018	2.41716
hsa-miR-27b	1.02E-03	6.33E-05	4.345336	1.4514	2.42269
hsa-miR-27b	1.05E-03	6.78E-05	4.324447	1.3849	2.405948
hsa-miR-27b	1.05E-03	7.28E-05	4.303446	1.3182	2.399966
hsa-miR-27b	1.05E-03	7.54E-05	4.293049	1.2853	2.39812
hsa-miR-27b	2.35E-03	2.77E-04	3.895375	0.0553	2.769597
hsa-miR-26b	8.89E-02	3.14E-02	2.209975	-4.2682	1.580262
hsa-miR-26b	1.03E-01	3.74E-02	2.135212	-4.4172	1.541311
hsa-miR-26a	4.18E-02	1.12E-02	2.628965	-3.3573	1.501223
hsa-miR-24-1*	7.15E-04	3.38E-05	4.530455	2.0466	2.299003
hsa-miR-24-1*	7.84E-04	4.03E-05	4.479	1.88	2.499743
hsa-miR-24-1*	1.25E-03	1.21E-04	4.14963	0.8348	2.289638
hsa-miR-23b	1.05E-03	7.57E-05	4.291629	1.2808	2.511477
hsa-miR-23b	1.05E-03	7.82E-05	4.281844	1.2498	2.530443
hsa-miR-23b	1.06E-03	8.35E-05	4.262121	1.1875	2.513339
hsa-miR-23b	1.08E-03	8.89E-05	4.243344	1.1283	2.483066
hsa-miR-23b	1.13E-03	9.87E-05	4.211781	1.0291	2.620661
hsa-miR-23b	1.13E-03	1.04E-04	4.196398	0.9809	2.456697
hsa-miR-23b	1.13E-03	1.05E-04	4.194068	0.9736	2.448701
hsa-miR-23b	1.16E-03	1.10E-04	4.180211	0.9302	2.451388
hsa-miR-23b	1.16E-03	1.10E-04	4.178547	0.925	2.457905
hsa-miR-23b	1.17E-03	1.12E-04	4.174639	0.9128	2.467665
hsa-miR-23b	1.21E-03	1.17E-04	4.160922	0.87	2.445011
hsa-miR-23b	1.27E-03	1.25E-04	4.140894	0.8076	2.430738
hsa-miR-23b	1.27E-03	1.26E-04	4.137587	0.7973	2.449157
hsa-miR-23b	1.31E-03	1.31E-04	4.12583	0.7607	2.425069
hsa-miR-23b	1.33E-03	1.34E-04	4.118613	0.7383	2.431312
hsa-miR-23b	1.51E-03	1.64E-04	4.056562	0.5465	2.704514
hsa-miR-221	4.57E-02	1.25E-02	2.585144	-3.4583	1.698537
hsa-miR-221	5.98E-02	1.86E-02	2.427548	-3.8109	1.649216
hsa-miR-221	6.64E-02	2.15E-02	2.369698	-3.9358	1.591325
hsa-miR-221	6.75E-02	2.20E-02	2.360398	-3.9557	1.539665
hsa-miR-221	6.84E-02	2.24E-02	2.352871	-3.9717	1.709009
hsa-miR-221	7.13E-02	2.36E-02	2.331305	-4.0175	1.505908
hsa-miR-221	7.15E-02	2.38E-02	2.326684	-4.0272	1.529319

hsa-miR-221	7.77E-02	2.64E-02	2.283501	-4.1176	1.556503
hsa-miR-221	8.40E-02	2.92E-02	2.241102	-4.2049	1.585805
hsa-miR-214	1.02E-03	6.35E-05	4.34443	1.4485	2.835438
hsa-miR-214	1.03E-03	6.45E-05	4.339773	1.4337	2.972057
hsa-miR-214	1.05E-03	7.19E-05	4.307171	1.3301	2.843497
hsa-miR-214	1.05E-03	7.76E-05	4.284236	1.2574	2.811937
hsa-miR-214	1.05E-03	7.91E-05	4.278708	1.2399	2.847831
hsa-miR-214	1.05E-03	8.03E-05	4.2741	1.2254	2.804516
hsa-miR-214	1.07E-03	8.58E-05	4.254198	1.1625	2.84275
hsa-miR-214	1.07E-03	8.60E-05	4.253543	1.1605	2.83326
hsa-miR-214	1.10E-03	9.40E-05	4.226474	1.0752	2.875281
hsa-miR-214	1.13E-03	1.01E-04	4.20621	1.0116	2.957051
hsa-miR-214	1.13E-03	1.01E-04	4.205154	1.0083	2.925236
hsa-miR-214	1.23E-03	1.19E-04	4.156172	0.8552	2.888848
hsa-miR-214	1.27E-03	1.26E-04	4.137572	0.7972	2.914621
hsa-miR-214	1.31E-03	1.32E-04	4.124339	0.7561	2.888099
hsa-miR-214	1.33E-03	1.35E-04	4.117861	0.736	2.859381
hsa-miR-214	1.41E-03	1.50E-04	4.085551	0.6359	2.862172
hsa-miR-20a*	1.14E-01	4.25E-02	2.078364	-4.5276	1.533156
hsa-miR-205	5.41E-08	2.23E-10	-7.813646	13.5324	-6.035633
hsa-miR-205	1.12E-07	5.09E-10	-7.589614	12.734	-5.468426
hsa-miR-204	4.26E-03	5.97E-04	3.651655	-0.6663	3.678198
hsa-miR-203	7.06E-04	3.31E-05	-4.536653	2.0667	-2.883104
hsa-miR-203	1.48E-03	1.59E-04	-4.067358	0.5798	-2.68355
hsa-miR-203	1.57E-03	1.71E-04	-4.043966	0.5077	-2.685294
hsa-miR-203	2.98E-03	3.72E-04	-3.802426	-0.223	-2.513072
hsa-miR-203	3.21E-03	4.17E-04	-3.766704	-0.329	-2.44862
hsa-miR-203	3.58E-03	4.74E-04	-3.725757	-0.4497	-2.575182
hsa-miR-203	3.81E-03	5.22E-04	-3.694896	-0.5403	-2.454415
hsa-miR-203	5.70E-03	8.47E-04	-3.538396	-0.9924	-2.505521
hsa-miR-203	5.77E-03	8.65E-04	-3.531607	-1.0117	-2.471119
hsa-miR-203	7.29E-03	1.13E-03	-3.443195	-1.2616	-2.417053
hsa-miR-203	8.43E-03	1.36E-03	-3.3828	-1.43	-2.403972
hsa-miR-200b*	9.76E-04	5.57E-05	-4.383272	1.5724	-2.192422
hsa-miR-200b*	1.13E-03	9.97E-05	-4.208841	1.0199	-2.033885
hsa-miR-200b*	6.92E-03	1.06E-03	-3.465056	-1.2002	-2.12937
hsa-miR-200b	1.15E-04	2.59E-06	-5.265381	4.503	-2.253171
hsa-miR-200b	1.42E-04	3.55E-06	-5.1769	4.2006	-2.182754
hsa-miR-200b	1.42E-04	3.63E-06	-5.170867	4.18	-2.191008
hsa-miR-200b	1.46E-04	3.80E-06	-5.158394	4.1375	-2.216388
hsa-miR-200b	1.47E-04	3.91E-06	-5.149855	4.1085	-2.183533
hsa-miR-200b	1.64E-04	4.52E-06	-5.109144	3.9701	-2.179142
hsa-miR-200b	2.96E-04	1.01E-05	-4.880631	3.2003	-2.140895
hsa-miR-200b	5.83E-04	2.39E-05	-4.63255	2.3794	-2.01034

hsa-miR-200b	6.23E-04	2.75E-05	-4.591299	2.2445	-2.003192
hsa-miR-200b	6.32E-04	2.84E-05	-4.58213	2.2146	-2.003496
hsa-miR-200a*	1.13E-05	5.60E-08	-6.319394	8.195	-3.509104
hsa-miR-200a*	2.89E-05	4.52E-07	-5.749456	6.1813	-3.30919
hsa-miR-200a*	4.35E-05	6.98E-07	-5.629987	5.7638	-3.04478
hsa-miR-200a*	9.15E-04	4.97E-05	-4.417261	1.6813	-2.665038
hsa-miR-200a	9.76E-04	5.65E-05	-4.378875	1.5584	-2.015141
hsa-miR-200a	1.05E-03	7.59E-05	-4.290773	1.2781	-2.010584
hsa-miR-200a	1.07E-03	8.72E-05	-4.249114	1.1465	-2.000524
hsa-miR-199b-5p	7.67E-04	3.91E-05	4.487807	1.9085	3.157549
hsa-miR-199b-5p	8.13E-04	4.30E-05	4.459596	1.8174	3.295318
hsa-miR-199b-5p	9.76E-04	5.59E-05	4.382158	1.5689	3.199582
hsa-miR-199b-5p	9.85E-04	5.91E-05	4.36579	1.5166	3.181438
hsa-miR-199b-5p	9.85E-04	6.01E-05	4.36039	1.4993	3.15713
hsa-miR-199b-5p	1.03E-03	6.50E-05	4.3372	1.4255	3.26486
hsa-miR-199b-5p	1.05E-03	7.19E-05	4.30707	1.3297	3.147219
hsa-miR-199b-5p	1.07E-03	8.69E-05	4.250162	1.1498	3.151959
hsa-miR-199b-5p	1.10E-03	9.30E-05	4.229777	1.0856	3.296424
hsa-miR-199b-5p	1.13E-03	1.04E-04	4.194889	0.9761	3.116649
hsa-miR-199b-5p	1.13E-03	1.05E-04	4.194484	0.9749	3.238275
hsa-miR-199b-5p	1.15E-03	1.08E-04	4.184386	0.9433	3.098567
hsa-miR-199b-5p	1.26E-03	1.23E-04	4.144395	0.8185	3.240427
hsa-miR-199b-5p	1.38E-03	1.46E-04	4.093469	0.6604	3.53329
hsa-miR-199b-5p	2.62E-03	3.16E-04	3.854323	-0.0681	3.313799
hsa-miR-199a-5p	1.08E-03	8.86E-05	4.2446	1.1323	2.963491
hsa-miR-199a-5p	1.13E-03	1.02E-04	4.200975	0.9952	2.86713
hsa-miR-199a-5p	1.13E-03	1.04E-04	4.194768	0.9758	2.86181
hsa-miR-199a-5p	1.26E-03	1.23E-04	4.145363	0.8215	2.850378
hsa-miR-199a-5p	1.36E-03	1.42E-04	4.100742	0.6829	2.822637
hsa-miR-199a-5p	1.43E-03	1.53E-04	4.079469	0.6171	2.79332
hsa-miR-199a-5p	1.70E-03	1.88E-04	4.015508	0.4204	2.75154
hsa-miR-199a-5p	1.88E-03	2.11E-04	3.979339	0.3098	2.875994
hsa-miR-199a-5p	2.08E-03	2.36E-04	3.944519	0.2039	2.927027
hsa-miR-199a-5p	2.08E-03	2.37E-04	3.943741	0.2016	2.958403
hsa-miR-199a-5p	2.67E-03	3.24E-04	3.846058	-0.0928	2.722203
hsa-miR-199a-5p	2.92E-03	3.58E-04	3.815019	-0.1855	2.793394
hsa-miR-199a-5p	2.97E-03	3.70E-04	3.80453	-0.2168	2.847011
hsa-miR-199a-5p	3.08E-03	3.91E-04	3.786687	-0.2698	3.233942
hsa-miR-199a-5p	3.66E-03	4.93E-04	3.713411	-0.486	3.278034
hsa-miR-199a-5p	5.44E-03	7.93E-04	3.559907	-0.9309	3.250844
hsa-miR-199a-3p	9.42E-04	5.16E-05	4.406154	1.6457	2.800512
hsa-miR-199a-3p	9.85E-04	5.90E-05	4.366113	1.5176	2.794476
hsa-miR-199a-3p	1.04E-03	6.60E-05	4.332824	1.4115	2.792669
hsa-miR-199a-3p	1.05E-03	6.75E-05	4.325892	1.3895	2.794922

hsa-miR-199a-3p	1.05E-03	7.28E-05	4.303508	1.3184	2.792328
hsa-miR-199a-3p	1.05E-03	7.29E-05	4.303167	1.3174	2.76694
hsa-miR-199a-3p	1.05E-03	7.65E-05	4.288471	1.2708	2.743717
hsa-miR-199a-3p	1.05E-03	7.77E-05	4.283689	1.2557	2.754817
hsa-miR-199a-3p	1.05E-03	7.91E-05	4.278557	1.2394	2.758932
hsa-miR-199a-3p	1.05E-03	8.07E-05	4.272464	1.2202	2.741261
hsa-miR-199a-3p	1.06E-03	8.40E-05	4.260636	1.1828	2.775951
hsa-miR-199a-3p	1.07E-03	8.49E-05	4.25736	1.1725	2.762768
hsa-miR-199a-3p	1.07E-03	8.51E-05	4.256457	1.1697	2.748775
hsa-miR-199a-3p	2.25E-03	2.61E-04	3.913932	0.1113	3.050997
hsa-miR-199a-3p	2.56E-03	3.04E-04	3.865782	-0.0337	3.068285
hsa-miR-199a-3p	2.96E-03	3.68E-04	3.806368	-0.2113	3.146072
hsa-miR-196b	2.95E-03	3.64E-04	3.809646	-0.2015	2.650818
hsa-miR-196b	3.16E-03	4.08E-04	3.773243	-0.3096	2.73387
hsa-miR-196b	3.19E-03	4.13E-04	3.769254	-0.3214	2.650166
hsa-miR-196b	3.60E-03	4.82E-04	3.720626	-0.4648	2.673536
hsa-miR-196b	3.64E-03	4.88E-04	3.716464	-0.477	2.47655
hsa-miR-196b	3.68E-03	4.98E-04	3.709694	-0.4969	2.734634
hsa-miR-196b	3.68E-03	4.99E-04	3.709595	-0.4972	2.653666
hsa-miR-196b	3.79E-03	5.18E-04	3.697386	-0.533	2.689467
hsa-miR-196b	3.88E-03	5.36E-04	3.686165	-0.5658	2.431484
hsa-miR-196b	3.88E-03	5.38E-04	3.685416	-0.568	2.430359
hsa-miR-196b	3.98E-03	5.53E-04	3.676413	-0.5943	2.688378
hsa-miR-196b	4.90E-03	7.01E-04	3.599772	-0.8165	2.547108
hsa-miR-196b	4.90E-03	7.03E-04	3.598822	-0.8192	2.468688
hsa-miR-196b	5.47E-03	7.99E-04	3.557203	-0.9387	2.548994
hsa-miR-196b	5.52E-03	8.13E-04	3.5517	-0.9544	2.504008
hsa-miR-196b	7.06E-03	1.09E-03	3.456666	-1.2238	2.569118
hsa-miR-195	2.31E-05	1.28E-07	6.095538	7.4005	3.5281
hsa-miR-195	2.31E-05	1.47E-07	6.057179	7.2648	3.497744
hsa-miR-195	2.31E-05	1.69E-07	6.018591	7.1284	3.568193
hsa-miR-195	2.31E-05	1.79E-07	6.003047	7.0735	3.462958
hsa-miR-195	2.31E-05	1.84E-07	5.996239	7.0494	3.458596
hsa-miR-195	2.31E-05	2.08E-07	5.962748	6.9312	3.465683
hsa-miR-195	2.31E-05	2.11E-07	5.958794	6.9173	3.497818
hsa-miR-195	2.31E-05	2.45E-07	5.91804	6.7736	3.641941
hsa-miR-195	2.31E-05	2.51E-07	5.910641	6.7476	3.613125
hsa-miR-195	2.31E-05	2.58E-07	5.903567	6.7226	3.645202
hsa-miR-195	2.31E-05	2.58E-07	5.903157	6.7212	3.480643
hsa-miR-195	2.31E-05	2.66E-07	5.894965	6.6924	3.636366
hsa-miR-195	2.33E-05	2.95E-07	5.866968	6.5938	3.632398
hsa-miR-195	2.33E-05	3.03E-07	5.859148	6.5663	3.656972
hsa-miR-195	2.33E-05	3.35E-07	5.831759	6.4701	3.631321
hsa-miR-195	2.72E-05	4.03E-07	5.781552	6.2938	3.657968

hsa-miR-183	5.44E-04	2.13E-05	-4.6662	2.4897	-2.76531
hsa-miR-183	6.58E-04	3.03E-05	-4.562431	2.1505	-2.712378
hsa-miR-183	7.97E-04	4.16E-05	-4.469403	1.8491	-2.576787
hsa-miR-183	9.85E-04	6.00E-05	-4.360901	1.501	-2.612221
hsa-miR-183	1.05E-03	6.95E-05	-4.317134	1.3617	-2.640136
hsa-miR-183	1.05E-03	7.27E-05	-4.303818	1.3194	-2.573601
hsa-miR-183	1.12E-03	9.60E-05	-4.220409	1.0562	-2.52747
hsa-miR-183	1.03E-02	1.72E-03	-3.302388	-1.6512	-2.699138
hsa-miR-183	3.22E-02	8.06E-03	-2.753624	-3.0627	-2.280852
hsa-miR-152	4.10E-04	1.50E-05	4.767068	2.8225	2.681952
hsa-miR-145*	5.83E-04	2.34E-05	4.638077	2.3975	3.411296
hsa-miR-145*	5.89E-04	2.47E-05	4.622401	2.3462	3.259305
hsa-miR-145*	1.04E-03	6.65E-05	4.330348	1.4037	3.638321
hsa-miR-145	3.69E-04	1.31E-05	4.805273	2.9492	3.529882
hsa-miR-145	4.12E-04	1.52E-05	4.762295	2.8067	3.439829
hsa-miR-145	5.21E-04	1.98E-05	4.687024	2.5582	3.419397
hsa-miR-145	5.27E-04	2.04E-05	4.678285	2.5295	3.537617
hsa-miR-145	5.45E-04	2.15E-05	4.662662	2.4781	3.425174
hsa-miR-145	5.83E-04	2.36E-05	4.635859	2.3902	3.484947
hsa-miR-145	6.52E-04	2.95E-05	4.570705	2.1774	3.440897
hsa-miR-145	6.56E-04	3.00E-05	4.56607	2.1623	3.400531
hsa-miR-145	7.06E-04	3.29E-05	4.538806	2.0737	3.451134
hsa-miR-145	7.16E-04	3.46E-05	4.524022	2.0257	3.348853
hsa-miR-145	7.16E-04	3.47E-05	4.522999	2.0224	3.406042
hsa-miR-145	7.28E-04	3.63E-05	4.510092	1.9806	3.45698
hsa-miR-145	7.37E-04	3.70E-05	4.504204	1.9615	3.361717
hsa-miR-145	7.50E-04	3.80E-05	4.496605	1.9369	3.345072
hsa-miR-145	1.05E-03	7.71E-05	4.286167	1.2635	3.704853
hsa-miR-145	1.06E-03	8.32E-05	4.263175	1.1909	3.725648
hsa-miR-143	1.39E-04	3.37E-06	5.192139	4.2526	3.445394
hsa-miR-143	2.08E-04	6.16E-06	5.021461	3.6733	3.388836
hsa-miR-143	2.08E-04	6.43E-06	5.009146	3.6318	3.366629
hsa-miR-143	2.08E-04	6.48E-06	5.007238	3.6253	3.341758
hsa-miR-143	2.08E-04	6.49E-06	5.006822	3.6239	3.363404
hsa-miR-143	2.33E-04	7.52E-06	4.964868	3.4827	3.325029
hsa-miR-143	2.33E-04	7.66E-06	4.959506	3.4646	3.387763
hsa-miR-143	2.42E-04	8.07E-06	4.944554	3.4144	3.311191
hsa-miR-140-5p	1.23E-04	2.88E-06	5.235974	4.4023	2.197935
hsa-miR-140-5p	1.52E-04	4.11E-06	5.135698	4.0603	2.197296
hsa-miR-140-5p	1.66E-04	4.65E-06	5.101372	3.9437	2.185884
hsa-miR-140-5p	1.83E-04	5.19E-06	5.069976	3.8373	2.111012
hsa-miR-140-5p	1.88E-04	5.41E-06	5.058106	3.7971	2.157625
hsa-miR-140-5p	1.90E-04	5.53E-06	5.051835	3.7759	2.176226
hsa-miR-140-5p	2.32E-04	7.35E-06	4.971343	3.5044	2.188602

hsa-miR-140-5p	2.33E-04	7.58E-06	4.962558	3.4749	2.149812
hsa-miR-140-5p	2.95E-04	9.96E-06	4.884509	3.2133	2.114042
hsa-miR-140-5p	3.15E-04	1.10E-05	4.857107	3.1218	2.134614
hsa-miR-140-5p	3.15E-04	1.10E-05	4.855592	3.1167	2.144421
hsa-miR-140-5p	3.83E-04	1.39E-05	4.789435	2.8966	2.136694
hsa-miR-140-5p	5.21E-04	1.99E-05	4.684787	2.5508	2.130105
hsa-miR-140-5p	5.89E-04	2.46E-05	4.624017	2.3514	2.226147
hsa-miR-140-5p	6.23E-04	2.77E-05	4.589273	2.2379	2.29174
hsa-miR-140-5p	1.57E-03	1.71E-04	4.044339	0.5089	2.303423
hsa-miR-140-3p	1.73E-03	1.91E-04	4.010104	0.4038	2.018462
hsa-miR-140-3p	1.85E-03	2.07E-04	3.985955	0.33	1.982043
hsa-miR-140-3p	2.04E-03	2.29E-04	3.953805	0.2321	1.965727
hsa-miR-140-3p	2.05E-03	2.32E-04	3.950289	0.2214	1.949859
hsa-miR-140-3p	2.19E-03	2.53E-04	3.923198	0.1393	1.977199
hsa-miR-140-3p	2.28E-03	2.66E-04	3.90747	0.0918	1.969032
hsa-miR-140-3p	2.40E-03	2.85E-04	3.886587	0.0288	1.92645
hsa-miR-140-3p	2.61E-03	3.12E-04	3.857715	-0.0579	1.912603
hsa-miR-136	2.31E-05	1.59E-07	6.035824	7.1893	3.12092
hsa-miR-135b	6.03E-04	2.63E-05	-4.604015	2.286	-3.417126
hsa-miR-135b	1.05E-03	7.22E-05	-4.305875	1.326	-2.921705
hsa-miR-135b	1.05E-03	7.62E-05	-4.28977	1.2749	-2.90218
hsa-miR-135b	1.05E-03	7.68E-05	-4.287426	1.2675	-2.945579
hsa-miR-135b	1.05E-03	7.98E-05	-4.275949	1.2312	-2.815989
hsa-miR-135b	1.06E-03	8.20E-05	-4.26761	1.2049	-2.777673
hsa-miR-135b	1.13E-03	9.69E-05	-4.217492	1.047	-2.718382
hsa-miR-135b	1.13E-03	1.04E-04	-4.196124	0.98	-2.8425
hsa-miR-135b	1.14E-03	1.06E-04	-4.190396	0.9621	-2.716756
hsa-miR-135b	1.36E-03	1.38E-04	-4.109595	0.7104	-2.695124
hsa-miR-135b	1.36E-03	1.40E-04	-4.106179	0.6998	-2.6942
hsa-miR-135b	1.36E-03	1.41E-04	-4.103759	0.6923	-2.705733
hsa-miR-135b	1.40E-03	1.48E-04	-4.08927	0.6474	-2.681903
hsa-miR-135b	1.51E-03	1.63E-04	-4.058968	0.5539	-2.702212
hsa-miR-135b	2.32E-03	2.73E-04	-3.900089	0.0695	-2.576923
hsa-miR-135b	2.95E-03	3.65E-04	-3.808593	-0.2047	-2.620982
hsa-miR-135a*	2.08E-04	6.49E-06	-5.00669	3.6235	-3.392195
hsa-miR-135a*	3.69E-04	1.32E-05	-4.803927	2.9447	-2.732912
hsa-miR-135a*	1.13E-03	9.81E-05	-4.213892	1.0357	-2.529884
hsa-miR-132*	3.10E-03	3.96E-04	3.783056	-0.2805	1.574876
hsa-miR-132*	6.03E-03	9.13E-04	3.513683	-1.0627	1.929516
hsa-miR-132*	1.44E-02	2.75E-03	3.141332	-2.0838	2.038358
hsa-miR-132	1.03E-02	1.74E-03	3.299449	-1.6593	1.810872
hsa-miR-132	1.13E-02	1.96E-03	3.258803	-1.7697	1.790335
hsa-miR-132	1.16E-02	2.03E-03	3.245923	-1.8045	1.809205
hsa-miR-132	1.18E-02	2.08E-03	3.237543	-1.8271	1.812181

hsa-miR-132	1.18E-02	2.08E-03	3.237483	-1.8273	1.750983
hsa-miR-132	1.19E-02	2.12E-03	3.231937	-1.8422	1.784468
hsa-miR-132	1.28E-02	2.35E-03	3.195475	-1.94	1.808139
hsa-miR-132	1.34E-02	2.51E-03	3.173221	-1.9993	1.736143
hsa-miR-132	1.65E-02	3.32E-03	3.075875	-2.2554	1.870289
hsa-miR-132	2.29E-02	5.01E-03	2.928414	-2.6328	2.022962
hsa-miR-127-3p	3.78E-03	5.13E-04	3.700401	-0.5241	3.2228
hsa-miR-127-3p	4.33E-03	6.11E-04	3.644327	-0.6876	3.353788
hsa-miR-126	1.29E-02	2.38E-03	3.191135	-1.9516	1.516397
hsa-miR-126	1.34E-02	2.51E-03	3.173641	-1.9982	1.518989
hsa-miR-126	1.45E-02	2.79E-03	3.13663	-2.0962	1.514972
hsa-miR-126	1.46E-02	2.83E-03	3.131763	-2.109	1.512317
hsa-miR-126	1.47E-02	2.87E-03	3.126185	-2.1237	1.525937
hsa-miR-126	1.68E-02	3.41E-03	3.06582	-2.2816	1.538143
hsa-miR-126	1.79E-02	3.70E-03	3.037493	-2.3549	1.507357
hsa-miR-126	1.91E-02	3.99E-03	3.010148	-2.4252	1.521527
hsa-miR-126	2.62E-02	6.15E-03	2.85361	-2.8191	1.540071
hsa-miR-126	2.76E-02	6.63E-03	2.826107	-2.8868	1.722931
hsa-miR-126	9.21E-02	3.28E-02	2.192464	-4.3035	1.939886
hsa-miR-126	1.07E-01	3.91E-02	2.115906	-4.455	1.948347
hsa-miR-125b-2*	4.78E-02	1.34E-02	2.557279	-3.5219	1.780825
hsa-miR-125b-2*	5.34E-02	1.58E-02	2.493594	-3.6652	1.898562
hsa-miR-125b	1.68E-02	3.42E-03	3.064864	-2.284	2.288053
hsa-miR-125b	1.69E-02	3.47E-03	3.060458	-2.2955	2.3331
hsa-miR-125b	1.86E-02	3.86E-03	3.022202	-2.3943	2.264475
hsa-miR-125b	1.86E-02	3.87E-03	3.021309	-2.3966	2.275082
hsa-miR-125b	2.01E-02	4.25E-03	2.988183	-2.4814	2.236249
hsa-miR-125b	2.02E-02	4.27E-03	2.98605	-2.4868	2.235584
hsa-miR-125b	2.11E-02	4.55E-03	2.963617	-2.5439	2.231998
hsa-miR-125b	2.18E-02	4.72E-03	2.950234	-2.5778	2.214991
hsa-miR-125b	2.24E-02	4.89E-03	2.937165	-2.6107	2.207963
hsa-miR-125b	2.40E-02	5.41E-03	2.900977	-2.7015	2.255835
hsa-miR-125b	2.43E-02	5.52E-03	2.893394	-2.7204	2.353281
hsa-miR-125b	2.43E-02	5.56E-03	2.89106	-2.7263	2.266032
hsa-miR-125b	2.50E-02	5.79E-03	2.876218	-2.7632	2.245203
hsa-miR-125b	2.61E-02	6.11E-03	2.856173	-2.8128	2.242828
hsa-miR-125b	2.64E-02	6.22E-03	2.849612	-2.829	2.211325
hsa-miR-125b	2.82E-02	6.84E-03	2.814604	-2.9149	2.192884
hsa-miR-10b	1.19E-02	2.10E-03	3.234064	-1.8365	2.240798
hsa-miR-10b	1.23E-02	2.21E-03	3.217751	-1.8804	2.284041
hsa-miR-10b	1.25E-02	2.28E-03	3.207072	-1.909	2.167075
hsa-miR-10b	1.31E-02	2.42E-03	3.186507	-1.9639	2.161368
hsa-miR-10b	1.33E-02	2.47E-03	3.178421	-1.9855	2.288075
hsa-miR-10b	1.34E-02	2.51E-03	3.173795	-1.9978	2.15294

hsa-miR-10b	1.37E-02	2.58E-03	3.16317	-2.026	2.246729
hsa-miR-10b	1.39E-02	2.64E-03	3.155911	-2.0452	2.246101
hsa-miR-10b	1.43E-02	2.73E-03	3.143451	-2.0782	2.288633
hsa-miR-10b	1.45E-02	2.78E-03	3.137298	-2.0944	2.135598
hsa-miR-10b	1.45E-02	2.80E-03	3.135064	-2.1003	2.142655
hsa-miR-10b	1.47E-02	2.86E-03	3.127956	-2.1191	2.139287
hsa-miR-10b	1.47E-02	2.88E-03	3.125978	-2.1243	2.272166
hsa-miR-10b	1.48E-02	2.91E-03	3.121331	-2.1365	2.116744
hsa-miR-10b	2.18E-02	4.73E-03	2.949356	-2.58	2.491319
hsa-miR-101	7.32E-03	1.14E-03	3.441128	-1.2674	1.692327
hsa-miR-101	8.43E-03	1.35E-03	3.383212	-1.4289	1.648727
hsa-miR-101	8.88E-03	1.44E-03	3.363413	-1.4836	1.629671
hsa-miR-101	9.07E-03	1.47E-03	3.354587	-1.508	1.564886
hsa-miR-101	9.11E-03	1.49E-03	3.351719	-1.5159	1.598533
hsa-miR-101	9.33E-03	1.53E-03	3.341567	-1.5439	1.593737
hsa-miR-101	9.33E-03	1.54E-03	3.340944	-1.5456	1.570912
hsa-miR-101	9.79E-03	1.62E-03	3.322286	-1.5968	1.680613
hsa-miR-101	9.84E-03	1.63E-03	3.319794	-1.6036	1.654126
hsa-miR-101	1.00E-02	1.67E-03	3.312508	-1.6236	1.605062
hsa-miR-101	1.04E-02	1.75E-03	3.29593	-1.6689	1.5984
hsa-miR-101	1.05E-02	1.80E-03	3.288035	-1.6904	1.637214
hsa-miR-101	1.10E-02	1.88E-03	3.272587	-1.7324	1.551569
hsa-miR-101	1.10E-02	1.90E-03	3.268827	-1.7426	1.680407
hsa-miR-101	1.18E-02	2.07E-03	3.239038	-1.8231	1.658807
hsa-miR-101	1.48E-02	2.91E-03	3.122235	-2.1341	1.637841
hsa-miR-100	1.36E-02	2.56E-03	3.166393	-2.0174	2.595684
hsa-miR-100	2.02E-02	4.29E-03	2.984198	-2.4916	2.525392
hsa-miR-100	2.03E-02	4.31E-03	2.98294	-2.4948	2.507411
hsa-miR-100	2.06E-02	4.40E-03	2.975572	-2.5135	2.464667
hsa-miR-100	2.14E-02	4.62E-03	2.958209	-2.5576	2.479062
hsa-miR-100	2.24E-02	4.88E-03	2.938418	-2.6076	2.448901
hsa-miR-100	2.31E-02	5.09E-03	2.92324	-2.6458	2.733968
hsa-miR-100	2.32E-02	5.13E-03	2.919822	-2.6544	2.453304
hsa-miR-100	2.32E-02	5.18E-03	2.916422	-2.6629	2.751434
hsa-miR-100	2.39E-02	5.38E-03	2.903083	-2.6963	2.689173
hsa-miR-100	2.40E-02	5.42E-03	2.899919	-2.7042	2.774586
hsa-miR-100	2.61E-02	6.13E-03	2.855245	-2.8151	2.72721
hsa-miR-100	2.67E-02	6.31E-03	2.844596	-2.8413	2.726579
hsa-miR-100	2.70E-02	6.42E-03	2.838073	-2.8574	2.699173
hsa-miR-100	2.85E-02	6.97E-03	2.807606	-2.932	2.789604
hsa-let-7c	4.72E-02	1.31E-02	2.56617	-3.5017	1.950153
hsa-let-7c	4.78E-02	1.34E-02	2.558204	-3.5198	1.93759
hsa-let-7c	5.05E-02	1.45E-02	2.527438	-3.5894	1.643228
hsa-let-7c	5.09E-02	1.47E-02	2.523367	-3.5986	1.949649

hsa-let-7c	5.13E-02	1.48E-02	2.519518	-3.6072	1.897737
hsa-let-7c	5.34E-02	1.58E-02	2.494616	-3.663	1.623935
hsa-let-7c	5.37E-02	1.60E-02	2.488733	-3.6761	1.900841
hsa-let-7c	5.39E-02	1.61E-02	2.486955	-3.68	1.616021
hsa-let-7c	5.39E-02	1.61E-02	2.485636	-3.6829	1.625902
hsa-let-7c	5.43E-02	1.63E-02	2.481295	-3.6926	1.620282
hsa-let-7c	5.56E-02	1.68E-02	2.469251	-3.7193	1.623894
hsa-let-7c	5.59E-02	1.69E-02	2.46702	-3.7242	2.100843
hsa-let-7c	5.60E-02	1.70E-02	2.465498	-3.7276	1.886725
hsa-let-7c	5.65E-02	1.71E-02	2.460991	-3.7375	1.612253
hsa-let-7c	5.66E-02	1.72E-02	2.459064	-3.7418	1.880387
hsa-let-7c	5.84E-02	1.79E-02	2.443455	-3.7761	1.617576
hsa-let-7b	3.44E-02	8.69E-03	2.725095	-3.131	1.524046
hsa-let-7b	3.94E-02	1.03E-02	2.66175	-3.2808	1.50066
hsa-let-7b	4.10E-02	1.08E-02	2.640998	-3.3293	1.535758
hsa-let-7b	4.10E-02	1.09E-02	2.640495	-3.3305	1.530075
hsa-let-7b	1.05E-01	3.83E-02	2.124791	-4.4376	1.899971
hsa-let-7b	1.18E-01	4.48E-02	2.055732	-4.5708	1.75516
