# Supplementary material for: Establishment and Comprehensive Analysis of Underlying microRNA-mRNA Interactive Networks in Ovarian Cancer
Source: J Oncol. 2022 Mar 10;2022:5120342. doi: 10.1155/2022/5120342 (PMC8930263; doi:10.1155/2022/5120342)
Supplement: Supplementary Materials — Table S1. DEMs between OC and normal tissue from the GSE25405 dataset. Table S2. DEMs between OC and normal tissue from the GSE119055 dataset. Table S3. Target genes of DEMs predicted by miRNet. [file 5120342.f1.zip › 5120342.f1/Supplementary Table S2.pdf]

Table S2 DEMs between OC and normal tissue from GSE119055 dataset.

ID	adj.P.Val	P.Value	t	B	logFC
hsa-miR-99a-5p	0.011829	2.62E-05	6.58072	2.9253	2.127703
hsa-miR-99a-3p	0.022911	8.01E-05	5.837002	1.8406	3.785004
hsa-miR-98-5p	0.2669	1.54E-02	2.823751	-3.0375	1.557868
hsa-miR-934	0.325009	2.71E-02	-2.51546	-3.8751	-2.52713
hsa-miR-874-5p	0.078966	1.14E-03	4.244582	-0.6224	2.43035
hsa-miR-708-5p	0.013362	4.09E-05	6.277831	2.5027	3.122317
hsa-miR-6723-5p	0.1695	5.39E-03	-3.38832	-2.0623	-2.42216
hsa-miR-660-5p	0.023796	9.48E-05	5.729706	1.7034	2.48161
hsa-miR-660-3p	0.011829	2.71E-05	6.556187	2.8133	3.480528
hsa-miR-654-3p	0.383023	3.89E-02	2.317597	-3.8949	3.127298
hsa-miR-642a-3p	0.140645	3.25E-03	-3.66336	-1.6142	-2.02163
hsa-miR-628-3p	0.316991	2.53E-02	2.552819	-3.4933	1.664421
hsa-miR-615-3p	0.2669	1.54E-02	2.821168	-3.0458	2.127695
hsa-miR-548q	0.023796	1.03E-04	5.678125	1.5999	2.304032
hsa-miR-543	0.261657	1.45E-02	2.853659	-2.9957	3.153474
hsa-miR-532-5p	0.076361	9.68E-04	4.336811	-0.5435	1.944603
hsa-miR-532-3p	0.023796	9.83E-05	5.706529	1.6692	2.568303
hsa-miR-513c-5p	0.340455	2.96E-02	2.467663	-3.6672	4.437059
hsa-miR-513b-5p	0.38603	4.06E-02	2.295267	-3.9454	3.842998
hsa-miR-513a-5p	0.36407	3.44E-02	2.386324	-3.8072	4.477551
hsa-miR-509-3p	0.411574	4.63E-02	2.221305	-4.1339	4.62585
hsa-miR-509-3-5p	0.402003	4.46E-02	2.241988	-4.0652	4.707724
hsa-miR-508-3p	0.312108	2.42E-02	2.577358	-3.4854	4.293856
hsa-miR-505-3p	0.108772	2.31E-03	3.851319	-1.2788	1.806257
hsa-miR-504-5p	0.161861	4.68E-03	3.464389	-1.9397	2.318871
hsa-miR-502-5p	0.005562	4.86E-06	7.803046	4.3501	4.220506
hsa-miR-502-3p	0.025447	1.30E-04	5.529679	1.4021	2.235492
hsa-miR-501-5p	0.09579	1.59E-03	4.057075	-0.9396	1.911532
hsa-miR-501-3p	0.005843	9.98E-06	7.264446	3.7804	2.672396
hsa-miR-500b-3p	0.011829	2.84E-05	6.523299	2.7627	3.440582
hsa-miR-500a-5p	0.162716	4.87E-03	3.442686	-2.0416	1.515641
hsa-miR-500a-3p	0.023796	1.04E-04	5.670539	1.6156	2.226168
hsa-miR-497-5p	0.1445	3.79E-03	3.579159	-1.8323	2.337917
hsa-miR-495-3p	0.150495	4.08E-03	3.539195	-1.8182	3.923412
hsa-miR-487b-3p	0.38749	4.16E-02	2.280856	-4.0332	2.160252
hsa-miR-455-5p	0.122677	2.68E-03	3.768365	-1.4227	3.236765
hsa-miR-4524a-3p	0.030077	1.84E-04	5.314938	1.061	1.856192
hsa-miR-4510	0.028978	1.58E-04	5.407681	1.2168	2.464702
hsa-miR-449c-5p	0.295417	2.09E-02	-2.65671	-3.6874	-2.63206
hsa-miR-449b-5p	0.272916	1.81E-02	-2.73578	-3.5845	-2.2105
hsa-miR-449a	0.201414	7.35E-03	-3.22037	-2.6601	-2.74449

hsa-miR-4458	0.08489	1.32E-03	4.162756	-0.8197	1.61307
hsa-miR-4429	0.040929	2.95E-04	5.028653	0.6222	1.948372
hsa-miR-4417	0.211403	8.57E-03	-3.13813	-2.5292	-2.14245
hsa-miR-433-3p	0.428951	4.92E-02	2.187548	-4.0977	2.562136
hsa-miR-4329	0.09993	1.81E-03	3.984486	-1.0731	1.637764
hsa-miR-4324	0.005843	1.02E-05	7.246864	3.6851	4.726414
hsa-miR-429	0.126484	2.82E-03	-3.74073	-1.7211	-3.01086
hsa-miR-4269	0.158552	4.44E-03	3.493607	-1.8933	3.569062
hsa-miR-424-5p	0.351658	3.26E-02	2.415993	-3.7348	3.279547
hsa-miR-424-3p	0.333979	2.83E-02	2.493528	-3.7112	2.261384
hsa-miR-411-5p	0.319729	2.59E-02	2.541053	-3.5512	3.326144
hsa-miR-3926	0.057651	5.55E-04	4.65689	0.0467	3.235637
hsa-miR-383-5p	0.000351	7.67E-08	11.51657	8.1192	7.695929
hsa-miR-381-5p	0.18575	6.20E-03	3.312631	-2.2217	3.01916
hsa-miR-381-3p	0.212613	8.79E-03	3.12454	-2.554	3.781094
hsa-miR-379-5p	0.383023	3.85E-02	2.324491	-3.9794	2.289794
hsa-miR-378i	0.241254	1.21E-02	-2.95408	-2.8476	-2.19316
hsa-miR-378f	0.201634	7.45E-03	-3.21337	-2.4004	-2.1877
hsa-miR-378e	0.1695	5.34E-03	-3.39276	-2.0778	-2.33076
hsa-miR-378c	0.222888	9.89E-03	-3.06066	-2.6886	-2.31143
hsa-miR-378a-5p	0.169681	5.53E-03	-3.37453	-2.0891	-2.62196
hsa-miR-378a-3p	0.211403	8.23E-03	-3.15981	-2.573	-2.4937
hsa-miR-377-3p	0.292101	2.05E-02	2.667854	-3.3888	2.404405
hsa-miR-376c-3p	0.227322	1.03E-02	3.039557	-2.7255	3.829461
hsa-miR-374b-5p	0.10839	2.27E-03	3.858974	-1.2615	2.749153
hsa-miR-3687	0.088892	1.42E-03	-4.1214	-0.8298	-2.43347
hsa-miR-362-5p	0.082463	1.23E-03	4.203332	-0.7265	1.850856
hsa-miR-362-3p	0.013145	3.45E-05	6.392489	2.6163	4.058282
hsa-miR-361-3p	0.169681	5.48E-03	3.379006	-2.0834	2.308541
hsa-miR-34c-5p	0.097041	1.65E-03	-4.03536	-1.1075	-2.44601
hsa-miR-34c-3p	0.105981	2.09E-03	-3.90703	-1.3518	-2.80471
hsa-miR-34a-3p	0.316991	2.54E-02	2.551409	-3.5163	2.102366
hsa-miR-339-3p	0.211403	8.30E-03	3.155159	-2.5309	1.509005
hsa-miR-337-5p	0.387422	4.11E-02	2.288135	-3.9836	2.484417
hsa-miR-331-5p	0.251135	1.35E-02	2.892152	-2.9127	1.604186
hsa-miR-329-3p	0.241254	1.17E-02	2.968552	-2.8249	3.32615
hsa-miR-328-3p	0.025447	1.34E-04	5.513776	1.3661	2.538118
hsa-miR-320e	0.024122	1.16E-04	5.601815	1.5124	1.966142
hsa-miR-3200-3p	0.1695	5.16E-03	-3.41153	-2.1988	-2.15438
hsa-miR-31-5p	0.222888	9.83E-03	-3.06382	-2.6822	-3.81873
hsa-miR-31-3p	0.1695	5.41E-03	-3.38649	-2.2659	-3.07748
hsa-miR-30e-3p	0.237213	1.13E-02	2.990578	-2.7672	2.335312
hsa-miR-30c-1-3p	0.14992	4.03E-03	3.545681	-1.7919	2.043385
hsa-miR-29c-5p	0.161381	4.59E-03	3.475594	-1.9162	2.659821

hsa-miR-29c-3p	0.005668	6.20E-06	7.618622	4.1836	3.126603
hsa-miR-29b-2-5p	0.288221	2.00E-02	2.681876	-3.3128	1.594104
hsa-miR-299-5p	0.196344	6.83E-03	3.260598	-2.3034	3.997679
hsa-miR-299-3p	0.261324	1.45E-02	2.856471	-2.9924	2.651127
hsa-miR-26b-5p	0.02981	1.76E-04	5.342757	1.1211	2.334825
hsa-miR-26b-3p	0.013362	4.03E-05	6.287328	2.501	2.431247
hsa-miR-224-5p	0.076361	8.93E-04	-4.38295	-0.4678	-3.25674
hsa-miR-221-5p	0.343979	3.06E-02	2.449904	-3.6607	1.681308
hsa-miR-214-5p	0.196344	6.82E-03	3.260879	-2.3247	3.489026
hsa-miR-214-3p	0.369338	3.55E-02	2.368176	-4.0823	1.647537
hsa-miR-21-3p	0.005562	3.66E-06	-8.02385	4.5477	-3.52398
hsa-miR-20a-3p	0.1445	3.59E-03	3.609266	-1.8449	1.691462
hsa-miR-204-5p	0.080637	1.18E-03	4.224287	-0.6621	4.495769
hsa-miR-202-3p	0.2669	1.63E-02	2.79041	-3.1636	5.589224
hsa-miR-200c-3p	0.406042	4.54E-02	-2.23316	-4.1357	-3.88958
hsa-miR-200b-5p	0.05105	4.35E-04	-4.79848	0.2574	-5.32181
hsa-miR-200b-3p	0.161381	4.57E-03	-3.47808	-1.9193	-4.60104
hsa-miR-200a-5p	0.076361	9.74E-04	-4.3332	-0.6111	-4.17093
hsa-miR-200a-3p	0.10839	2.26E-03	-3.86292	-1.2724	-5.44383
hsa-miR-199b-3p	0.251135	1.36E-02	2.889584	-3.1902	2.291912
hsa-miR-199a-5p	0.197874	7.11E-03	3.238427	-2.5251	2.362391
hsa-miR-199a-3p	0.251135	1.36E-02	2.889584	-3.1902	2.291912
hsa-miR-195-5p	0.090265	1.46E-03	4.105158	-1.0293	2.339028
hsa-miR-195-3p	0.057651	5.44E-04	4.66855	0.0717	3.101318
hsa-miR-193a-3p	0.1149	2.46E-03	3.815475	-1.3529	1.673218
hsa-miR-18a-5p	0.097023	1.63E-03	-4.04266	-0.9704	-2.40491
hsa-miR-187-3p	0.00498	2.18E-06	-8.4378	5.2003	-5.21864
hsa-miR-186-5p	0.295852	2.10E-02	2.654243	-3.444	2.233384
hsa-miR-183-5p	0.014813	4.86E-05	-6.16337	2.3335	-4.81626
hsa-miR-183-3p	0.161861	4.71E-03	-3.46099	-2.1466	-2.18393
hsa-miR-182-5p	0.102548	1.95E-03	-3.94516	-1.1316	-4.42457
hsa-miR-154-5p	0.272916	1.81E-02	2.736491	-3.214	3.348579
hsa-miR-149-5p	0.238213	1.14E-02	2.984039	-2.7925	1.751568
hsa-miR-148a-3p	0.262176	1.48E-02	2.844172	-3.0828	1.972247
hsa-miR-1468-5p	0.076361	9.68E-04	4.336676	-0.5521	1.508062
hsa-miR-145-3p	0.216128	9.03E-03	3.110043	-2.5993	3.078528
hsa-miR-143-3p	0.251135	1.36E-02	2.890326	-3.2112	1.843361
hsa-miR-141-3p	0.255561	1.40E-02	-2.87276	-2.998	-5.48355
hsa-miR-140-5p	0.206494	7.67E-03	3.197346	-2.4354	2.880102
hsa-miR-140-3p	0.1445	3.65E-03	3.60002	-1.8547	1.532346
hsa-miR-138-1-3p	0.076361	9.28E-04	-4.3607	-0.4511	-2.16613
hsa-miR-135a-3p	0.144121	3.53E-03	3.618193	-1.6718	2.697433
hsa-miR-134-3p	0.211403	8.39E-03	3.149342	-2.5073	2.540057
hsa-miR-133b	0.067654	7.25E-04	4.502208	-0.2275	2.412372

hsa-miR-133a-3p	0.219474	9.26E-03	3.096163	-2.6136	3.040945
hsa-miR-132-5p	0.231617	1.06E-02	3.024503	-2.7643	2.304685
hsa-miR-130b-3p	0.057249	5.13E-04	-4.70209	0.1204	-2.22206
hsa-miR-130a-3p	0.059236	5.96E-04	4.615346	-0.0868	1.538921
hsa-miR-1294	0.024122	1.15E-04	5.609681	1.5249	2.430566
hsa-miR-1271-5p	0.02981	1.71E-04	5.360575	1.1484	2.662363
hsa-miR-125b-5p	0.030296	1.92E-04	5.288998	0.9381	1.624104
hsa-miR-125b-2-3p	0.210747	7.92E-03	3.180661	-2.4886	2.081519
hsa-miR-125b-1-3p	0.116207	2.52E-03	3.803665	-1.3596	3.407574
hsa-miR-125a-5p	0.076361	1.02E-03	4.308118	-0.6796	1.592608
hsa-miR-1247-5p	0.031561	2.07E-04	5.243302	0.9681	3.17416
hsa-miR-10b-5p	0.076361	1.00E-03	4.315815	-0.5815	2.246001
hsa-miR-10b-3p	0.2669	1.54E-02	2.823979	-3.0629	2.312795
hsa-miR-101-5p	0.005843	9.47E-06	7.302544	3.8003	2.886921
hsa-miR-101-3p	0.057651	5.32E-04	4.680754	0.0879	2.908892
hsa-miR-100-5p	0.0344	2.33E-04	5.171008	0.7944	2.308918
hsa-let-7e-3p	0.220869	9.46E-03	3.084449	-2.619	2.427944
