# Supplementary material for: Establishment and Comprehensive Analysis of Underlying microRNA-mRNA Interactive Networks in Ovarian Cancer
Source: J Oncol. 2022 Mar 10;2022:5120342. doi: 10.1155/2022/5120342 (PMC8930263; doi:10.1155/2022/5120342)
Supplement: Supplementary Materials — Table S1. DEMs between OC and normal tissue from the GSE25405 dataset. Table S2. DEMs between OC and normal tissue from the GSE119055 dataset. Table S3. Target genes of DEMs predicted by miRNet. [file 5120342.f1.zip › 5120342.f1/Supplementary Table S3.pdf]

Table S3 Target genes of DEMs predicted by miRNet.

ID	Gene
hsa-mir-199a-5p	ACVR1B
hsa-mir-199a-5p	JAG1
hsa-mir-199a-5p	APOE
hsa-mir-199a-5p	DDR1
hsa-mir-199a-5p	CAV1
hsa-mir-199a-5p	SERPINH1
hsa-mir-199a-5p	CD44
hsa-mir-199a-5p	CDH1
hsa-mir-199a-5p	CDH2
hsa-mir-199a-5p	CDK9
hsa-mir-199a-5p	CDKN1C
hsa-mir-199a-5p	CTSC
hsa-mir-199a-5p	RCC1
hsa-mir-199a-5p	CLTC
hsa-mir-199a-5p	CCR7
hsa-mir-199a-5p	COL19A1
hsa-mir-199a-5p	COX15
hsa-mir-199a-5p	CSNK2A1
hsa-mir-199a-5p	CTGF
hsa-mir-199a-5p	DDX3X
hsa-mir-199a-5p	SLC26A2
hsa-mir-199a-5p	E2F3
hsa-mir-199a-5p	EDN1
hsa-mir-199a-5p	ERBB2
hsa-mir-199a-5p	ERBB3
hsa-mir-199a-5p	ERN1
hsa-mir-199a-5p	ETS1
hsa-mir-199a-5p	ETS2
hsa-mir-199a-5p	EXTL3
hsa-mir-199a-5p	EZH2
hsa-mir-199a-5p	GATA6
hsa-mir-199a-5p	GM2A
hsa-mir-199a-5p	GSK3B
hsa-mir-199a-5p	HIF1A
hsa-mir-199a-5p	HK2
hsa-mir-199a-5p	HSPA5
hsa-mir-199a-5p	IKBKB
hsa-mir-199a-5p	ITGA3
hsa-mir-199a-5p	JUNB
hsa-mir-199a-5p	KRAS
hsa-mir-199a-5p	LDLR
hsa-mir-199a-5p	LIF

hsa-mir-199a-5p	SMAD3
hsa-mir-199a-5p	SMAD4
hsa-mir-199a-5p	MECP2
hsa-mir-199a-5p	MAP3K9
hsa-mir-199a-5p	MAP3K11
hsa-mir-199a-5p	ABCC1
hsa-mir-199a-5p	NAB2
hsa-mir-199a-5p	NDUFS2
hsa-mir-199a-5p	NFKB1
hsa-mir-199a-5p	PDE4D
hsa-mir-199a-5p	PIK3CD
hsa-mir-199a-5p	PIN1
hsa-mir-199a-5p	PODXL
hsa-mir-199a-5p	POLR2F
hsa-mir-199a-5p	PSG3
hsa-mir-199a-5p	PSG11
hsa-mir-199a-5p	PSMD9
hsa-mir-199a-5p	PTGS2
hsa-mir-199a-5p	QSOX1
hsa-mir-199a-5p	NECTIN1
hsa-mir-199a-5p	SLC8A1
hsa-mir-199a-5p	SMARCA2
hsa-mir-199a-5p	SNAI1
hsa-mir-199a-5p	SNAP25
hsa-mir-199a-5p	SNTB1
hsa-mir-199a-5p	SOX9
hsa-mir-199a-5p	SULT1E1
hsa-mir-199a-5p	TFDP2
hsa-mir-199a-5p	TGFB2
hsa-mir-199a-5p	TGFBR1
hsa-mir-199a-5p	TUBG1
hsa-mir-199a-5p	UNG
hsa-mir-199a-5p	VASP
hsa-mir-199a-5p	VEGFA
hsa-mir-199a-5p	WNT2
hsa-mir-199a-5p	ZNF195
hsa-mir-199a-5p	ZNF215
hsa-mir-199a-5p	PAX8
hsa-mir-199a-5p	AKAP17A
hsa-mir-199a-5p	FZD4
hsa-mir-199a-5p	FZD6
hsa-mir-199a-5p	MAP4K3
hsa-mir-199a-5p	BECN1
hsa-mir-199a-5p	LIN7A

hsa-mir-199a-5p	TSC22D1
hsa-mir-199a-5p	KL
hsa-mir-199a-5p	CRIPT
hsa-mir-199a-5p	MAFB
hsa-mir-199a-5p	OXSR1
hsa-mir-199a-5p	MED6
hsa-mir-199a-5p	TRIM10
hsa-mir-199a-5p	PIAS3
hsa-mir-199a-5p	VAV3
hsa-mir-199a-5p	RER1
hsa-mir-199a-5p	DDX19B
hsa-mir-199a-5p	ATF6
hsa-mir-199a-5p	RAB21
hsa-mir-199a-5p	PLXND1
hsa-mir-199a-5p	SIRT1
hsa-mir-199a-5p	POLA2
hsa-mir-199a-5p	RNF11
hsa-mir-199a-5p	GPR78
hsa-mir-199a-5p	RNF115
hsa-mir-199a-5p	RND1
hsa-mir-199a-5p	ZNF544
hsa-mir-199a-5p	SETD2
hsa-mir-199a-5p	TMOD2
hsa-mir-199a-5p	PDE11A
hsa-mir-199a-5p	ZDHHC9
hsa-mir-199a-5p	ZNF117
hsa-mir-199a-5p	SLC38A2
hsa-mir-199a-5p	LAX1
hsa-mir-199a-5p	VPS53
hsa-mir-199a-5p	DRAM1
hsa-mir-199a-5p	DNAJA4
hsa-mir-199a-5p	ZNF415
hsa-mir-199a-5p	CSGALNACT1
hsa-mir-199a-5p	PLGRKT
hsa-mir-199a-5p	CCNL1
hsa-mir-199a-5p	AGTRAP
hsa-mir-199a-5p	RIC8A
hsa-mir-199a-5p	C16orf58
hsa-mir-199a-5p	PLEKHG2
hsa-mir-199a-5p	WNK1
hsa-mir-199a-5p	CENPO
hsa-mir-199a-5p	PANK3
hsa-mir-199a-5p	PTCD2
hsa-mir-199a-5p	ZNF669

hsa-mir-199a-5p	C3orf36
hsa-mir-199a-5p	NAA15
hsa-mir-199a-5p	DDHD1
hsa-mir-199a-5p	ZNF611
hsa-mir-199a-5p	SESN2
hsa-mir-199a-5p	ZNF394
hsa-mir-199a-5p	DDI2
hsa-mir-199a-5p	ZBTB37
hsa-mir-199a-5p	CHRFAM7A
hsa-mir-199a-5p	ZNF468
hsa-mir-199a-5p	ZNF625
hsa-mir-199a-5p	ARHGAP12
hsa-mir-199a-5p	TMEM54
hsa-mir-199a-5p	TNFRSF13C
hsa-mir-199a-5p	SLC16A10
hsa-mir-199a-5p	ZNF440
hsa-mir-199a-5p	OSCP1
hsa-mir-199a-5p	CHCHD4
hsa-mir-199a-5p	XRRA1
hsa-mir-199a-5p	A2ML1
hsa-mir-199a-5p	CEP120
hsa-mir-199a-5p	SNRNP48
hsa-mir-199a-5p	TBC1D21
hsa-mir-199a-5p	ZFP1
hsa-mir-199a-5p	ZNF846
hsa-mir-199a-5p	ZNF791
hsa-mir-199a-5p	ZNF525
hsa-mir-199a-5p	PLPP4
hsa-mir-199a-5p	ZNF584
hsa-mir-199a-5p	DYNAP
hsa-mir-199a-5p	ZNF844
hsa-mir-199a-5p	SLC27A1
hsa-mir-199a-5p	ZNF772
hsa-mir-199a-5p	C1orf226
hsa-mir-199a-5p	ZNF286B
hsa-mir-199a-5p	PSAPL1
hsa-mir-199a-3p	AKT1
hsa-mir-199a-3p	APOE
hsa-mir-199a-3p	CAV2
hsa-mir-199a-3p	CD44
hsa-mir-199a-3p	CDK7
hsa-mir-199a-3p	COL4A5
hsa-mir-199a-3p	COX10
hsa-mir-199a-3p	MAPK14

hsa-mir-199a-3p	CSNK1A1
hsa-mir-199a-3p	CSRP2
hsa-mir-199a-3p	E2F2
hsa-mir-199a-3p	ETS2
hsa-mir-199a-3p	FGF2
hsa-mir-199a-3p	FLT1
hsa-mir-199a-3p	FXN
hsa-mir-199a-3p	MTOR
hsa-mir-199a-3p	FUT4
hsa-mir-199a-3p	GNAS
hsa-mir-199a-3p	HGF
hsa-mir-199a-3p	HLA-B
hsa-mir-199a-3p	FOXA2
hsa-mir-199a-3p	ID2
hsa-mir-199a-3p	IGF1
hsa-mir-199a-3p	FOXK2
hsa-mir-199a-3p	ITGA3
hsa-mir-199a-3p	JUNB
hsa-mir-199a-3p	KDR
hsa-mir-199a-3p	KRT7
hsa-mir-199a-3p	MECP2
hsa-mir-199a-3p	MAP3K4
hsa-mir-199a-3p	MET
hsa-mir-199a-3p	KMT2A
hsa-mir-199a-3p	CDK17
hsa-mir-199a-3p	PKM
hsa-mir-199a-3p	MAPK1
hsa-mir-199a-3p	MAPK8
hsa-mir-199a-3p	MAPK9
hsa-mir-199a-3p	PTGS2
hsa-mir-199a-3p	SMARCA2
hsa-mir-199a-3p	SOD2
hsa-mir-199a-3p	STK11
hsa-mir-199a-3p	TFAM
hsa-mir-199a-3p	UBE2V1
hsa-mir-199a-3p	UCK2
hsa-mir-199a-3p	VEGFA
hsa-mir-199a-3p	DDX39B
hsa-mir-199a-3p	MLF2
hsa-mir-199a-3p	STC2
hsa-mir-199a-3p	UNC5C
hsa-mir-199a-3p	SGPL1
hsa-mir-199a-3p	SYNGR2
hsa-mir-199a-3p	QKI

hsa-mir-199a-3p	GFPT2
hsa-mir-199a-3p	MED6
hsa-mir-199a-3p	TSPAN3
hsa-mir-199a-3p	PAK4
hsa-mir-199a-3p	YAP1
hsa-mir-199a-3p	PNRC1
hsa-mir-199a-3p	FICD
hsa-mir-199a-3p	ZHX1
hsa-mir-199a-3p	TAB2
hsa-mir-199a-3p	PHF8
hsa-mir-199a-3p	GGA3
hsa-mir-199a-3p	BLOC1S6
hsa-mir-199a-3p	RGS17
hsa-mir-199a-3p	GOLIM4
hsa-mir-199a-3p	CCDC59
hsa-mir-199a-3p	SOCS7
hsa-mir-199a-3p	TMED5
hsa-mir-199a-3p	HSD17B12
hsa-mir-199a-3p	VTA1
hsa-mir-199a-3p	LUC7L2
hsa-mir-199a-3p	MTRF1L
hsa-mir-199a-3p	DDIT4
hsa-mir-199a-3p	ARL15
hsa-mir-199a-3p	PAQR5
hsa-mir-199a-3p	FKBP14
hsa-mir-199a-3p	CEP55
hsa-mir-199a-3p	C11orf57
hsa-mir-199a-3p	DNAJA4
hsa-mir-199a-3p	ETNK1
hsa-mir-199a-3p	SLC30A6
hsa-mir-199a-3p	ATF7IP
hsa-mir-199a-3p	CCNL1
hsa-mir-199a-3p	CREBZF
hsa-mir-199a-3p	MCCC2
hsa-mir-199a-3p	ZNF106
hsa-mir-199a-3p	VPS33A
hsa-mir-199a-3p	WNK2
hsa-mir-199a-3p	TAF1D
hsa-mir-199a-3p	EFHD2
hsa-mir-199a-3p	NOL10
hsa-mir-199a-3p	ZNF585B
hsa-mir-199a-3p	NACC1
hsa-mir-199a-3p	SMYD4
hsa-mir-199a-3p	WDR17

hsa-mir-199a-3p	TMEM18
hsa-mir-199a-3p	OCIAD2
hsa-mir-199a-3p	LIN54
hsa-mir-199a-3p	CCDC80
hsa-mir-199a-3p	TMEM161B
hsa-mir-199a-3p	ZXDB
hsa-mir-199a-3p	TOR1AIP2
hsa-mir-199a-3p	ZNF384
hsa-mir-199a-3p	ZDHHC24
hsa-mir-199a-3p	MCOLN2
hsa-mir-199a-3p	CNEP1R1
hsa-mir-199a-3p	LINC00346
hsa-mir-199a-3p	RGMB
hsa-mir-199a-3p	ACER2
hsa-mir-199a-3p	RBM43
hsa-mir-199a-3p	TMEM189
hsa-mir-199a-3p	TMEM189-UBE2V1
hsa-mir-199a-3p	C7orf55-LUC7L2
hsa-mir-140-5p	ADA
hsa-mir-140-5p	ALDH1A1
hsa-mir-140-5p	PRDM1
hsa-mir-140-5p	DST
hsa-mir-140-5p	CAPN1
hsa-mir-140-5p	CEBPD
hsa-mir-140-5p	CSK
hsa-mir-140-5p	DNMT1
hsa-mir-140-5p	E2F3
hsa-mir-140-5p	EP300
hsa-mir-140-5p	ESR2
hsa-mir-140-5p	FEN1
hsa-mir-140-5p	FGF2
hsa-mir-140-5p	FGF9
hsa-mir-140-5p	GALC
hsa-mir-140-5p	GATA6
hsa-mir-140-5p	HOXD12
hsa-mir-140-5p	IGF1R
hsa-mir-140-5p	IGFBP5
hsa-mir-140-5p	LAMC1
hsa-mir-140-5p	LDLR
hsa-mir-140-5p	MMP13
hsa-mir-140-5p	CYTB
hsa-mir-140-5p	MYO6
hsa-mir-140-5p	NDUFA2
hsa-mir-140-5p	SEPT2

hsa-mir-140-5p	PAX6
hsa-mir-140-5p	PDGFRA
hsa-mir-140-5p	PIN1
hsa-mir-140-5p	KLK10
hsa-mir-140-5p	RALA
hsa-mir-140-5p	SOX2
hsa-mir-140-5p	SOX4
hsa-mir-140-5p	SOX9
hsa-mir-140-5p	STAT1
hsa-mir-140-5p	TGFBR1
hsa-mir-140-5p	VEGFA
hsa-mir-140-5p	VEZF1
hsa-mir-140-5p	CUBN
hsa-mir-140-5p	FXR1
hsa-mir-140-5p	BRAP
hsa-mir-140-5p	FZD6
hsa-mir-140-5p	STK24
hsa-mir-140-5p	ADAM9
hsa-mir-140-5p	RIOK3
hsa-mir-140-5p	PHACTR2
hsa-mir-140-5p	HDAC4
hsa-mir-140-5p	TSC22D2
hsa-mir-140-5p	ZC3H11A
hsa-mir-140-5p	MED13
hsa-mir-140-5p	BCL2L11
hsa-mir-140-5p	PPIE
hsa-mir-140-5p	PRPF8
hsa-mir-140-5p	IGF2BP1
hsa-mir-140-5p	RAB10
hsa-mir-140-5p	ZNF652
hsa-mir-140-5p	CNKSR2
hsa-mir-140-5p	RPRD2
hsa-mir-140-5p	LARP1
hsa-mir-140-5p	MMD
hsa-mir-140-5p	DNPEP
hsa-mir-140-5p	TSPAN12
hsa-mir-140-5p	ARIH1
hsa-mir-140-5p	SNORD12C
hsa-mir-140-5p	RPUSD2
hsa-mir-140-5p	RAB30
hsa-mir-140-5p	OSTM1
hsa-mir-140-5p	GLRX5
hsa-mir-140-5p	HDAC7
hsa-mir-140-5p	TM7SF3

hsa-mir-140-5p	FGFRL1
hsa-mir-140-5p	ZCCHC2
hsa-mir-140-5p	MRPS10
hsa-mir-140-5p	MEG3
hsa-mir-140-5p	YOD1
hsa-mir-140-5p	STRADB
hsa-mir-140-5p	CAMK2N1
hsa-mir-140-5p	SMURF1
hsa-mir-140-5p	LYRM2
hsa-mir-140-5p	GALNT16
hsa-mir-140-5p	GPR107
hsa-mir-140-5p	SPCS3
hsa-mir-140-5p	SNX16
hsa-mir-140-5p	AIDA
hsa-mir-140-5p	CASD1
hsa-mir-140-5p	ZNF426
hsa-mir-140-5p	EFHD2
hsa-mir-140-5p	HMGN5
hsa-mir-140-5p	PANK3
hsa-mir-140-5p	KATNBL1
hsa-mir-140-5p	ACTR5
hsa-mir-140-5p	YIPF4
hsa-mir-140-5p	PLEKHA8
hsa-mir-140-5p	SMCR8
hsa-mir-140-5p	SIRPA
hsa-mir-140-5p	SPRED1
hsa-mir-140-5p	IFNLR1
hsa-mir-140-5p	TMED4
hsa-mir-140-5p	RNF149
hsa-mir-140-5p	ZDHHC21
hsa-mir-140-5p	LIN28B
hsa-mir-455-5p	APLP2
hsa-mir-455-5p	TRIM23
hsa-mir-455-5p	RHOH
hsa-mir-455-5p	RUNX1T1
hsa-mir-455-5p	CD36
hsa-mir-455-5p	CDKN1B
hsa-mir-455-5p	CRKL
hsa-mir-455-5p	DDX3X
hsa-mir-455-5p	DYRK1A
hsa-mir-455-5p	ERCC4
hsa-mir-455-5p	ETS2
hsa-mir-455-5p	HOXA1
hsa-mir-455-5p	KPNA3

hsa-mir-455-5p	MAP3K9
hsa-mir-455-5p	MYBL1
hsa-mir-455-5p	DRG1
hsa-mir-455-5p	OTX1
hsa-mir-455-5p	PCCA
hsa-mir-455-5p	PCCB
hsa-mir-455-5p	PIK3R1
hsa-mir-455-5p	PTPRB
hsa-mir-455-5p	REL
hsa-mir-455-5p	RPS6KB1
hsa-mir-455-5p	RPS14
hsa-mir-455-5p	SLC1A5
hsa-mir-455-5p	SOX11
hsa-mir-455-5p	UBA7
hsa-mir-455-5p	ZNF134
hsa-mir-455-5p	ZNF138
hsa-mir-455-5p	ZFAND5
hsa-mir-455-5p	SOCS3
hsa-mir-455-5p	TXNL1
hsa-mir-455-5p	QKI
hsa-mir-455-5p	PCLAF
hsa-mir-455-5p	RASSF2
hsa-mir-455-5p	G3BP1
hsa-mir-455-5p	VAV3
hsa-mir-455-5p	IPO7
hsa-mir-455-5p	ZNF460
hsa-mir-455-5p	CCNI
hsa-mir-455-5p	POLI
hsa-mir-455-5p	FKBP9
hsa-mir-455-5p	PLEKHA6
hsa-mir-455-5p	SEPHS1
hsa-mir-455-5p	RAB18
hsa-mir-455-5p	IGSF9B
hsa-mir-455-5p	FBXO28
hsa-mir-455-5p	ARC
hsa-mir-455-5p	MGRN1
hsa-mir-455-5p	NCSTN
hsa-mir-455-5p	PRKD2
hsa-mir-455-5p	LYPD3
hsa-mir-455-5p	ZNF544
hsa-mir-455-5p	MYLIP
hsa-mir-455-5p	ZNF354C
hsa-mir-455-5p	ZNF117
hsa-mir-455-5p	TRPV2

hsa-mir-455-5p	TRIM33
hsa-mir-455-5p	NUP54
hsa-mir-455-5p	PRR13
hsa-mir-455-5p	DDX4
hsa-mir-455-5p	BNC2
hsa-mir-455-5p	AHI1
hsa-mir-455-5p	PIWIL2
hsa-mir-455-5p	MTPAP
hsa-mir-455-5p	DARS2
hsa-mir-455-5p	DEPDC1B
hsa-mir-455-5p	NUFIP2
hsa-mir-455-5p	FAM160B1
hsa-mir-455-5p	ATP13A3
hsa-mir-455-5p	MOB3B
hsa-mir-455-5p	TMC7
hsa-mir-455-5p	DSN1
hsa-mir-455-5p	WDR26
hsa-mir-455-5p	KLHL15
hsa-mir-455-5p	GSG1
hsa-mir-455-5p	SLC9A7
hsa-mir-455-5p	UBASH3B
hsa-mir-455-5p	ZNF625
hsa-mir-455-5p	LYRM7
hsa-mir-455-5p	NT5C1B
hsa-mir-455-5p	MOGAT1
hsa-mir-455-5p	C15orf40
hsa-mir-455-5p	TMEM170A
hsa-mir-455-5p	TNFAIP8L1
hsa-mir-455-5p	PABPC4L
hsa-mir-455-5p	UBXN2B
hsa-mir-455-5p	LETM2
hsa-mir-455-5p	TRUB1
hsa-mir-455-5p	BCDIN3D
hsa-mir-455-5p	TCF23
hsa-mir-455-5p	CAMSAP1
hsa-mir-455-5p	DNAJC18
hsa-mir-455-5p	PATL1
hsa-mir-455-5p	YIPF6
hsa-mir-455-5p	ZNF772
hsa-mir-455-5p	ZBTB34
hsa-mir-455-5p	FAM229B
hsa-mir-455-5p	NT5C1B-RDH14
hsa-mir-140-3p	ABL2
hsa-mir-140-3p	ACVR2B

hsa-mir-140-3p	ADARB1
hsa-mir-140-3p	AHCY
hsa-mir-140-3p	AMFR
hsa-mir-140-3p	BCAT1
hsa-mir-140-3p	KLF9
hsa-mir-140-3p	CAPN1
hsa-mir-140-3p	CAPZA1
hsa-mir-140-3p	CASP10
hsa-mir-140-3p	CD38
hsa-mir-140-3p	CDC25A
hsa-mir-140-3p	CDK6
hsa-mir-140-3p	AP2M1
hsa-mir-140-3p	CNN2
hsa-mir-140-3p	COL4A1
hsa-mir-140-3p	CLDN7
hsa-mir-140-3p	CRY2
hsa-mir-140-3p	EPB41
hsa-mir-140-3p	EPRS
hsa-mir-140-3p	ERCC1
hsa-mir-140-3p	ESR2
hsa-mir-140-3p	FANCA
hsa-mir-140-3p	FAU
hsa-mir-140-3p	FDFT1
hsa-mir-140-3p	FN1
hsa-mir-140-3p	FPGS
hsa-mir-140-3p	GDNF
hsa-mir-140-3p	GFPT1
hsa-mir-140-3p	GGT7
hsa-mir-140-3p	GPC1
hsa-mir-140-3p	MKNK2
hsa-mir-140-3p	GUK1
hsa-mir-140-3p	NCKAP1L
hsa-mir-140-3p	HMGN1
hsa-mir-140-3p	HMGN2
hsa-mir-140-3p	HNRNPA2B1
hsa-mir-140-3p	ITGA6
hsa-mir-140-3p	KCNMB1
hsa-mir-140-3p	KIF5A
hsa-mir-140-3p	TNPO1
hsa-mir-140-3p	LAMP2
hsa-mir-140-3p	LRPAP1
hsa-mir-140-3p	MAX
hsa-mir-140-3p	MAP3K3
hsa-mir-140-3p	MICB

hsa-mir-140-3p	MOBP
hsa-mir-140-3p	MYO6
hsa-mir-140-3p	NFYA
hsa-mir-140-3p	NRF1
hsa-mir-140-3p	FURIN
hsa-mir-140-3p	ATP8B1
hsa-mir-140-3p	PLCL1
hsa-mir-140-3p	MAPK8
hsa-mir-140-3p	RPL22
hsa-mir-140-3p	RPS24
hsa-mir-140-3p	CLIP1
hsa-mir-140-3p	ATXN1
hsa-mir-140-3p	CXCL6
hsa-mir-140-3p	SKI
hsa-mir-140-3p	SLC1A4
hsa-mir-140-3p	SLC2A5
hsa-mir-140-3p	SNTB2
hsa-mir-140-3p	CAPN15
hsa-mir-140-3p	SS18
hsa-mir-140-3p	TMBIM6
hsa-mir-140-3p	THRA
hsa-mir-140-3p	TPI1
hsa-mir-140-3p	TRPC4
hsa-mir-140-3p	UBE2V1
hsa-mir-140-3p	UBTF
hsa-mir-140-3p	UCK2
hsa-mir-140-3p	WEE1
hsa-mir-140-3p	ZNF207
hsa-mir-140-3p	SLC30A3
hsa-mir-140-3p	BTG2
hsa-mir-140-3p	PABPN1
hsa-mir-140-3p	SLC7A5
hsa-mir-140-3p	MKKS
hsa-mir-140-3p	NRIP1
hsa-mir-140-3p	ITGA10
hsa-mir-140-3p	STC2
hsa-mir-140-3p	PSMG1
hsa-mir-140-3p	KSR1
hsa-mir-140-3p	USP14
hsa-mir-140-3p	GCNT3
hsa-mir-140-3p	ZBTB22
hsa-mir-140-3p	GDF15
hsa-mir-140-3p	KIAA0040
hsa-mir-140-3p	GPRIN2

hsa-mir-140-3p	DAZAP2
hsa-mir-140-3p	POM121
hsa-mir-140-3p	AP5Z1
hsa-mir-140-3p	ZBTB40
hsa-mir-140-3p	SLC23A1
hsa-mir-140-3p	MAMLD1
hsa-mir-140-3p	ATP6AP2
hsa-mir-140-3p	AP3S2
hsa-mir-140-3p	FSTL3
hsa-mir-140-3p	EMC8
hsa-mir-140-3p	TUBA1B
hsa-mir-140-3p	ATP8A1
hsa-mir-140-3p	ZER1
hsa-mir-140-3p	TADA3
hsa-mir-140-3p	NEBL
hsa-mir-140-3p	MYL12A
hsa-mir-140-3p	NUP50
hsa-mir-140-3p	ZNF460
hsa-mir-140-3p	CD3EAP
hsa-mir-140-3p	SERINC3
hsa-mir-140-3p	TMED10
hsa-mir-140-3p	RAB31
hsa-mir-140-3p	NUDT21
hsa-mir-140-3p	UBE2C
hsa-mir-140-3p	TRIM31
hsa-mir-140-3p	XPOT
hsa-mir-140-3p	VASH1
hsa-mir-140-3p	ADGRL1
hsa-mir-140-3p	MLXIP
hsa-mir-140-3p	KIAA0907
hsa-mir-140-3p	ZNF423
hsa-mir-140-3p	NFASC
hsa-mir-140-3p	GRAMD4
hsa-mir-140-3p	TTLL12
hsa-mir-140-3p	DNAJC9
hsa-mir-140-3p	PDS5A
hsa-mir-140-3p	ANKRD12
hsa-mir-140-3p	CBX6
hsa-mir-140-3p	SUZ12
hsa-mir-140-3p	DDAH1
hsa-mir-140-3p	PPP1R15A
hsa-mir-140-3p	CADM1
hsa-mir-140-3p	PITPNB
hsa-mir-140-3p	IBTK

hsa-mir-140-3p	GAPVD1
hsa-mir-140-3p	SENP3
hsa-mir-140-3p	PITPNC1
hsa-mir-140-3p	PALD1
hsa-mir-140-3p	RACGAP1
hsa-mir-140-3p	PSAT1
hsa-mir-140-3p	TNPO2
hsa-mir-140-3p	ZBTB21
hsa-mir-140-3p	COMMD2
hsa-mir-140-3p	IER3IP1
hsa-mir-140-3p	PCYOX1
hsa-mir-140-3p	CTDSPL2
hsa-mir-140-3p	ESF1
hsa-mir-140-3p	ERRFI1
hsa-mir-140-3p	HEATR5B
hsa-mir-140-3p	MAP10
hsa-mir-140-3p	CDCA4
hsa-mir-140-3p	CDCA8
hsa-mir-140-3p	CCDC198
hsa-mir-140-3p	RPRD1A
hsa-mir-140-3p	SETD5
hsa-mir-140-3p	LRRC20
hsa-mir-140-3p	KIF21A
hsa-mir-140-3p	SERTAD4
hsa-mir-140-3p	CLDND1
hsa-mir-140-3p	SLC39A10
hsa-mir-140-3p	LYRM2
hsa-mir-140-3p	CBX8
hsa-mir-140-3p	ZNF490
hsa-mir-140-3p	NUFIP2
hsa-mir-140-3p	ERAP2
hsa-mir-140-3p	MARC1
hsa-mir-140-3p	IPPK
hsa-mir-140-3p	TBC1D15
hsa-mir-140-3p	USP46
hsa-mir-140-3p	MARCKSL1
hsa-mir-140-3p	DDA1
hsa-mir-140-3p	TMEM109
hsa-mir-140-3p	CORO7
hsa-mir-140-3p	C3orf52
hsa-mir-140-3p	SCD5
hsa-mir-140-3p	WDR26
hsa-mir-140-3p	INTS14
hsa-mir-140-3p	TMEM133

hsa-mir-140-3p	ARID5B
hsa-mir-140-3p	DDI2
hsa-mir-140-3p	BRMS1L
hsa-mir-140-3p	EBPL
hsa-mir-140-3p	AIFM2
hsa-mir-140-3p	TBRG1
hsa-mir-140-3p	SHANK3
hsa-mir-140-3p	ZNF518B
hsa-mir-140-3p	BOD1
hsa-mir-140-3p	CABLES1
hsa-mir-140-3p	CHRDL1
hsa-mir-140-3p	MTDH
hsa-mir-140-3p	ORMDL3
hsa-mir-140-3p	GLMP
hsa-mir-140-3p	FAM46B
hsa-mir-140-3p	SLC16A10
hsa-mir-140-3p	C11orf74
hsa-mir-140-3p	SLC24A4
hsa-mir-140-3p	TTC39C
hsa-mir-140-3p	FAM213B
hsa-mir-140-3p	FAM3D
hsa-mir-140-3p	VKORC1L1
hsa-mir-140-3p	GPR180
hsa-mir-140-3p	ZNF431
hsa-mir-140-3p	SPATA13
hsa-mir-140-3p	PXDC1
hsa-mir-140-3p	FOXK1
hsa-mir-140-3p	ZDHHC23
hsa-mir-140-3p	METRNL
hsa-mir-140-3p	NWD1
hsa-mir-140-3p	C17orf51
hsa-mir-140-3p	ACER2
hsa-mir-140-3p	NANOS1
hsa-mir-140-3p	EVX2
hsa-mir-140-3p	DRAXIN
hsa-mir-140-3p	STUM
hsa-mir-140-3p	GJB7
hsa-mir-140-3p	TMEM189
hsa-mir-140-3p	TMEM189-UBE2V1
hsa-mir-140-3p	IL17REL
hsa-mir-140-3p	TMEM236
hsa-mir-140-3p	TMEM78
hsa-mir-140-3p	TMED7-TICAM2
hsa-mir-140-3p	MTRNR2L5

hsa-mir-140-3p	C15orf38-AP3S2
hsa-mir-140-3p	BCL2L2-PABPN1
hsa-mir-429	XIAP
hsa-mir-429	ARHGDIA
hsa-mir-429	RERE
hsa-mir-429	ATP5G3
hsa-mir-429	ATP6V1E1
hsa-mir-429	AVPR1A
hsa-mir-429	BCL2
hsa-mir-429	CASP2
hsa-mir-429	SERPINH1
hsa-mir-429	CCNT2
hsa-mir-429	SEPT7
hsa-mir-429	CDKN1B
hsa-mir-429	CRKL
hsa-mir-429	CYP1A1
hsa-mir-429	DNMT1
hsa-mir-429	EP300
hsa-mir-429	EZH2
hsa-mir-429	ACSL4
hsa-mir-429	FKBP5
hsa-mir-429	GATA6
hsa-mir-429	GOT1
hsa-mir-429	MKNK2
hsa-mir-429	HIF1A
hsa-mir-429	HOXB5
hsa-mir-429	IGF2
hsa-mir-429	IL4
hsa-mir-429	IRS1
hsa-mir-429	JUN
hsa-mir-429	KRAS
hsa-mir-429	LMNB1
hsa-mir-429	M6PR
hsa-mir-429	DNAJB9
hsa-mir-429	MYB
hsa-mir-429	MYC
hsa-mir-429	PAK2
hsa-mir-429	PDPK1
hsa-mir-429	PKD1
hsa-mir-429	PLCG1
hsa-mir-429	PMAIP1
hsa-mir-429	MAPK7
hsa-mir-429	PTEN
hsa-mir-429	PTPN4

hsa-mir-429	PTPRD
hsa-mir-429	RBBP4
hsa-mir-429	SHC1
hsa-mir-429	SHOX2
hsa-mir-429	FSCN1
hsa-mir-429	SOX2
hsa-mir-429	SP1
hsa-mir-429	ELOC
hsa-mir-429	TCF7L2
hsa-mir-429	ZEB1
hsa-mir-429	TIMP2
hsa-mir-429	TPD52L1
hsa-mir-429	TUBB2A
hsa-mir-429	UBE2D1
hsa-mir-429	VEGFA
hsa-mir-429	MLLT10
hsa-mir-429	SHOC2
hsa-mir-429	YEATS4
hsa-mir-429	BAP1
hsa-mir-429	KLF11
hsa-mir-429	CDC14B
hsa-mir-429	STX16
hsa-mir-429	RTL8C
hsa-mir-429	QKI
hsa-mir-429	ONECUT2
hsa-mir-429	BAG4
hsa-mir-429	PHF14
hsa-mir-429	SECISBP2L
hsa-mir-429	RASSF2
hsa-mir-429	ZEB2
hsa-mir-429	BCL2L11
hsa-mir-429	MAMLD1
hsa-mir-429	DNAJB6
hsa-mir-429	ARPC3
hsa-mir-429	ABI2
hsa-mir-429	DLC1
hsa-mir-429	SEC23A
hsa-mir-429	NCOA2
hsa-mir-429	CELF1
hsa-mir-429	SEC24A
hsa-mir-429	WASF3
hsa-mir-429	FRS2
hsa-mir-429	MALT1
hsa-mir-429	RASSF8

hsa-mir-429	WDR37
hsa-mir-429	GPATCH8
hsa-mir-429	CAMSAP2
hsa-mir-429	PSD3
hsa-mir-429	ZFPM2
hsa-mir-429	OSTF1
hsa-mir-429	PABPC1
hsa-mir-429	SESN1
hsa-mir-429	KLHL20
hsa-mir-429	OSTM1
hsa-mir-429	TBK1
hsa-mir-429	CLEC2D
hsa-mir-429	ABT1
hsa-mir-429	NRBP1
hsa-mir-429	NGRN
hsa-mir-429	CRLF3
hsa-mir-429	WNT16
hsa-mir-429	CAB39
hsa-mir-429	C21orf91
hsa-mir-429	ERRFI1
hsa-mir-429	RIN2
hsa-mir-429	DDIT4
hsa-mir-429	UHRF1BP1
hsa-mir-429	INO80D
hsa-mir-429	TMEM70
hsa-mir-429	CDCA4
hsa-mir-429	VAC14
hsa-mir-429	N4BP2
hsa-mir-429	MBD5
hsa-mir-429	ERBIN
hsa-mir-429	BBX
hsa-mir-429	PPM1H
hsa-mir-429	NLGN4X
hsa-mir-429	NUFIP2
hsa-mir-429	KLHL42
hsa-mir-429	TXNDC16
hsa-mir-429	RAP2C
hsa-mir-429	C6orf47
hsa-mir-429	CACNG8
hsa-mir-429	ELMO2
hsa-mir-429	KIF13A
hsa-mir-429	ZMAT3
hsa-mir-429	PRRG4
hsa-mir-429	TAF1D

hsa-mir-429	ZFHX4
hsa-mir-429	SHCBP1
hsa-mir-429	ERMP1
hsa-mir-429	CLPB
hsa-mir-429	TRIM56
hsa-mir-429	TCF7L1
hsa-mir-429	PARD6B
hsa-mir-429	UNC119B
hsa-mir-429	MTDH
hsa-mir-429	TJAP1
hsa-mir-429	TP53INP1
hsa-mir-429	DCBLD2
hsa-mir-429	FAM216B
hsa-mir-429	DENND5B
hsa-mir-429	PPP1R18
hsa-mir-429	TUBB
hsa-mir-429	ZNF621
hsa-mir-429	TMEM119
hsa-mir-429	MALAT1
hsa-mir-429	GPX8
hsa-mir-429	ANKRD33B
